# Posthandling Spectral Information Enhancement for Single Cell Raman Molecular Mapping Analysis

**DOI:** 10.1021/acs.analchem.5c03915

**Published:** 2025-10-27

**Authors:** Ankit Raj, Nungnit Wattanavichean, Makoto Kawamukai, Tatsuyuki Yamamoto, Hiro-o Hamaguchi

**Affiliations:** † Department of Chemistry and Institute of Molecular Science, 34914 National Yang Ming Chiao Tung University, 1001 University Road, Hsinchu 300, Taiwan; ‡ Department of Chemistry, Faculty of Science, Gakushuin University, 1-5-1 Mejiro, Toshima, Tokyo 171-8588, Japan; § School of Materials Science and Innovation, Faculty of Science, 26685 Mahidol University, Phuttamonthon 4 Road, Salaya, Nakhon Pathom 73170, Thailand; ∥ Department of Life Sciences, 12938 Shimane University, 1060 Nishikawatsucho, Matsue, Shimane 690-8504, Japan

## Abstract

Biochemical analysis of living systems such as single cells benefits greatly from the label-free and low-invasive molecular mapping with Raman microspectroscopy. Sets of Raman spectra at different spatial points are analyzed to generate Raman molecular maps corresponding to specific chemical species. However, human error and subjective data analysis can be technical issues that limit interpretation and its validity. Here, we present an objective data analysis scheme for postprocessing large data sets of Raman spectra for molecular mapping of living cells. The process comprises three steps: (i) Denoising the spectral data set using low-rank approximation; (ii) obtaining an objective background from data points outside the target cell; (iii) subtracting the thus obtained background using Hypothetical Addition Multivariate Analysis with Numerical Differentiation (HAMAND) via an automatically determined coefficient. Through the present analysis, minor Raman peaks, as indiscernible as they are, can be identified and precisely mapped. We demonstrate a quantitative discussion of cellular components after extracting contributions only from a single target cell from a Raman mapping image where multiple cells or parts of other cells are present. This work opens an improved analysis workflow for accurate spectroscopic analysis of living cells with the advantage of identifying minor Raman peaks unambiguously.

## Graphical abstract

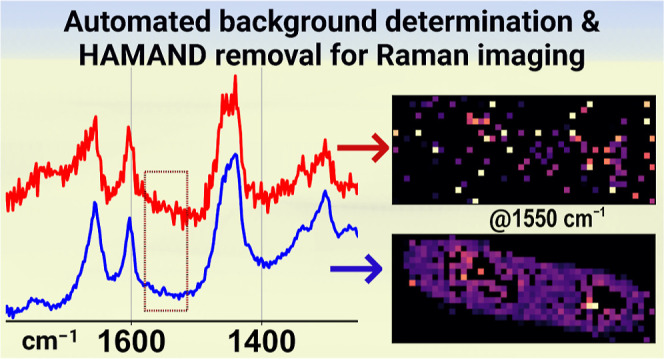

1

The physicochemical analysis of biological systems in the simplest living form starts with single cells. Various microscopic techniques have opened new avenues in understanding the complex biomachinery of cells through visualizing their structure and dynamics.^1,2^ Among those microscopic techniques, Raman microspectroscopy is a unique tool by providing chemically specific vibrational signatures in a label-free and low invasive manner.^3,4^ It has been actively used to study biological systems both in vitro and in vivo under physiological conditions, including single-cell Raman molecular mapping focusing on biochemical changes during cell-cycle.^5–9^ Analysis of mapping data has provided crucial information about the localization of chemical species, thus shedding new light on the partitioning of biomolecules in organelles and their roles before, during, and after cellular functions.^10–13^

However, Raman mapping data sets are often contaminated by the contribution of Raman signal and/or fluorescence from the substrate and/or cover glass with which cells are measured. This contamination as a spectral background tends to obscure the Raman spectra for further thorough analysis in complex ways. For example, a simple numerical subtraction of a background may result in negative features in the resultant spectra, and the background slope may affect unpredictably the band-fitting determination of area intensities. Although mathematical approaches such as multivariate curve resolution (MCR) have been employed to single out one or two components as background from the total data set, their process requires subjective choice of the number of spectral components, normalization coefficients^14–17^ and good initial guesses. Moreover, spectral mixing of the background with Raman spectra remains a problem affecting the obtained concentration profiles and eventual interpretation.^18^ Thus, objective determination of background and its automatic subtraction are important and indispensable steps preceding thorough spectral analysis in Raman molecular mapping.

Raman studies of a small number of single cells are not sufficient for understanding cellular biochemistry under a specific set of growth conditions. The dynamic nature of the cell cycle further complicates the situation. The low signal-to-noise ratio (SNR) of Raman spectra, constraint on laser power, and throughput of the spectrometer limit the number of cells measured. Typical experiments can only track tens of cells, a tiny fraction of the population available in the growth culture, restricting the generality of studies. There must be a push for multiplexing to maximize the number of cells measured to obtain larger and more conclusive data sets. Analysis of such large data sets in an automatic and objective manner is thus paramount.

With this motivation, we discuss a data postprocessing scheme developed for analyzing the Raman spectra of single cells with a view to quantitative analysis on the relative concentrations of cellular metabolites. We focus on an automatic and objective determination of background and its careful removal that enables a thorough spectral analysis, facilitating discussion of minor Raman bands. The processing scheme is demonstrated for Raman molecular mappings of *Schizosaccharomyces pombe*, a model eukaryotic cell popularly used for single-cell studies. Cells of *S. pombe* have a typical dimension of 5–10 μm, allowing for clear demarcation of cellular structures under a microscope with a high-magnification objective lens. Using a submicron laser spot, typically used in Raman imaging, fairly detailed Raman molecular maps are constructed. Although intense Raman peaks, such as those from lipids, proteins, nucleic acids, and more, are well established and frequently analyzed, we emphasize the next progress in such work: the successful identification and characterization of minor Raman bands, and our work focuses on this development via the proposed analysis scheme.

This article is structured as follows: Experiments and samples are described first. Results showing improvement in the Raman spectra and corresponding Raman maps with each step of the data analysis are introduced. Next, molecular maps of a few individual Raman signatures are discussed to demonstrate the effectiveness of the introduced post-processing analysis. Results for four cells are discussed to ascertain effectiveness over data sets of varying quality. Lastly, a brief comparison with results from other analysis workflows is shown.

## 2 Methods

### 2.1 Measurements and Samples

The Raman measurements were performed on a custom-developed Raman microspectrometer based on 632.8 nm excitation from a He–Ne laser, which was directed to an inverted microscope (Nikon Eclipse TE2000-U) for sample placement. The Raman optical setup was based on backscattering geometry with the excitation laser and Raman signal passing through a 100× microscope objective lens (Nikon Plan Fluor 100×/1.30 DIC H/N2). The Raman scattered light passed through a confocal optical setup before entering an imaging polychromator (Jobin-Yvon iHR320) and was detected by a CCD (Princeton Instruments, Spec-10). Lateral and axial spatial resolution were determined by the knife-edge method to be 0.3 μm and about 2.5 μm, respectively. Spectral coverage was from 586 to 715 nm, or from −1250 to 1800 cm^–1^ in relative wavenumbers with respect to the laser. A mercury lamp (Nikon Intensilight C-HGFI) was used with a band-pass filter to obtain fluorescence excitation at 469 ± 35 nm, which was directed to an inverted microscope via the same optical setup as the Raman excitation laser. Both the Raman scattered light and the fluorescence emission followed the same optical path to be detected simultaneously on the CCD. An optical image of the GFP fluorescence was acquired by using a band-pass filter transmitting 525 ± 39 nm with a Nikon DS-Ri1 camera unit. Laser power for Raman measurements on the sample was 1 mW.

The translation step size was 0.4 μm (using a piezo stage) with a Raman acquisition time of 2 s per point.

#### 2.1.1 Samples

As described earlier,^19^ the *S. pombe* cells measured in the present experiments were prepared by GFP-tagging of the Coq3 protein using pSLF272L-GFPS65A vector.^20^ The Coq3 protein is involved in the biosynthesis of coenzyme Q in the inner membrane of mitochondria.^21^ The GFP tag allows us to visualize mitochondria during the cell cycle^19^ via signal acquired from 586 to 608 nm (or from −1250 to −750 cm^–1^ in relative wavenumbers). Refer to Section SM1 in Supporting Information for additional details on measurements.

A total of four cells having distinct SNR ratios were analyzed using the proposed analysis scheme for rigorous testing. For brevity, the result for a cell is discussed in detail, while those for three other cells are given in the Supporting Information (see Section SM8). Refer to Table 1 for details on the mapped cells.

**Table 1:** Details of Living Cells Discussed in the Present Work

notation	cell culture duration (h)	pixels (*x*)	pixels (*y*)	cell area (μm^2^)	number of spectra	SNR of the 1443 cm^–1^ Raman peak^a^
C1^b^	24	21	28	47.6 ± 0.6	588	36.5
C2^c^	12	19	46	77.7 ± 0.6	874	26.1
C3^c^	12	26	28	65.7 ± 0.6	728	36.2
C4^c^	24	29	21	43.5 ± 0.6	609	5.4

a SNR computed after averaging the top 20 most intense spectra in the 2D data set (indicates the upper limit of the SNR in the 2D data set).

b Refer to the results section.

c Additional results presented in the Supporting Information.

### 2.2 Data Analysis Methodology

#### 2.2.1 Noise Reduction Using Low-Rank Reconstruction after Singular Value Decomposition (SVD)

Raman spectroscopic data contain noise from many different origins, such as detector readout, dark noise, and more. In order to improve the signal-to-noise ratio (SNR), a reconstruction of the original data set using a certain number of principal components obtained after SVD matrix decomposition was performed. Such a process for noise reduction has been used in spectroscopic data analysis^18,22^ and in general signal processing for some time.^23,24^

The operation of SVD on the original matrix *S*_*m*×*n*_ produces *U*Σ*V* ^*T*^ matrices, and a reconstruction of *S*_*m*×*n*_ while retaining only a few large *k* singular values results in a low-rank approximation of *S*_*m*×*n*_^*k*^.^25^ This removes the high-frequency noise-like components, which resemble the noise encountered in spectroscopic measurements. To minimize information loss and retain minor spectral features, we use the metric defined as the SNR of the spectral vectors (*U*_*i*_).^26^ The SNR was computed as $SNR\left(U_{i}\right)=\frac{\sigma \left(signal\right)}{\sigma \left(noise\right)}$ , where the signal is obtained by smoothing *U*_*i*_, hereby labeled as *U*_*i*_^*s*^, and the noise is extracted by subtracting the smoothed signal from the original, noise = *U*_*i*_ – *U*_*i*_^*s*^. Savitzky–Golay filter was used for smoothing.^27^ A value of SNR over 1 indicates the presence of spectral information in *U*_*i*_, and all spectral vectors such that SNR­(*U*_*i*_) > 1 were retained during reconstruction of the data set. This condition has been used in earlier work on identifying relevant spectral vectors in the analysis of Raman spectra of tissues.^26^ See Section SM2 in the Supporting Information for additional details.

### 2.3 Objective Determination of the Background Spectra

In a confocal setup, the Raman scattered photons are collected from a few micrometers in the axial or *z*-direction. Thus, when living cells are measured, the whole cell is captured along this direction. However, in addition to the living cell, the confocal volume also contains a glass substrate and the growth medium whose Raman signal is measured simultaneously. Hence, it becomes important to identify the background spectrum (the spectrum of the substrate and the growth medium), which should be subtracted from the raw data prior to further analysis.

For this, first, we devised a scheme to identify the spatial points from outside the cell based on the intensity of the Raman peak relative to the baseline. The peak-to-baseline intensity ratio, defined as $I_{x,y}\left(\nu {\;cm}^{-1}\right)=\left(\frac{p_{x,y}}{b_{x,y}}\right)$ from spatial coordinates (*x*,*y*) when considering a Raman band at ν cm^–1^, would be greater than unity. For a spectrum with no Raman band at ν cm^–1^, such a ratio would be equal to or less than one. A filter based on this principle allows one to separate out spatial points inside and outside the living cell, by targeting a Raman peak which is specific to the cell. This scheme provides an objective basis for identifying the spectra specific to the living cell in the Raman map and vice versa. In the present analysis, intensity for baseline and at the peak was determined by averaging three consecutive points in the peak-free region and at the peak top (identified by the user), respectively.

In the present analysis, Raman peaks at 1440 cm^–1^ from CH_2_-bend originating from lipids and proteins were used for the determination of points outside the cell. Spatial points with *I*_*x*,*y*_(1440 cm^–1^) having a value ≤1 were filtered out to retain spectra with a relatively flat background, which typically accounted for over 96% of all spectra from outside the cell. The corresponding Raman spectra from these spatial points were averaged to obtain the background signal, which was used in the next step.

Other Raman peaks present throughout the living cell, such as the ring-breathing mode of phenylalanine at 1003 cm^–1^ (from proteins), can also be used for the purpose, as demonstrated in Section SM4.

### 2.4 Subtraction of Background Spectrum Using Hypothetical Addition Multivariate Analysis with Numerical Differentiation (HAMAND)

During the Raman mapping of cells, the effective composition of the confocal volume changes due to the varying refractive index of the cell and the medium around it. This effect is significant near the periphery of the cell. However, the background spectrum is exclusively obtained from outside the cell (as discussed in the previous section). Hence, when subtracting this background spectrum ( B⃗ ) from the denoised spectrum ( ${\mathrm{R⃗}}_{i}$ ), it should not be assumed that the subtraction coefficient, *c*_*i*_, is unity in the operation, ${\mathrm{R⃗}}_{i}^{sub}={\mathrm{R⃗}}_{i}-\mathrm{B⃗}\times c_{i}$ .

In the present case, the magnitude of *c*_*i*_ was determined by using HAMAND analysis. Hypothetical addition multivariate analysis with numerical differentiation (HAMAND)^28,29^ combines the principle of the standard addition method with the numerical chemometric method for separating and quantifying a known target spectral component from a complex spectrum. This procedure has been applied for objective subtraction of minor components in Raman spectra of various chemical systems.^30,31^ HAMAND processes a number of numerically generated hypothetical model spectra (***S***_*j*_), which are obtained by adding the standard spectrum (the background spectrum in present work, B⃗ ) to the spectrum of an unknown sample (R⃗) with a range of generated coefficients (*c*_*j*_), expressed as $S_{j}=\mathrm{R⃗}+c_{j}\times \mathrm{B⃗}$ . This hypothetical set of spectra, ***S***_*j*_, is separated, via non-negative least-squares minimization, into spectral components and their corresponding concentration profiles: *W*_1_ (the spectrum corresponding to all the other substances except for the target), *W*_2_ (the target; in the present case, it is the background spectrum, which is known); the intensity profile of *H*_1_ (as constant) and *H*_2_ (which is linearly dependent on the hypothetically added coefficient representing concentration, *c*_*j*_). The calibration line, which is obtained by plotting the intensity profile *H*_2_ versus the hypothetically added coefficient, *c*_*j*_, determines the amount of the target spectrum already contained in the sample via the intercept. The underlying algorithm is visualized in Figure 1.

**Figure 1:**
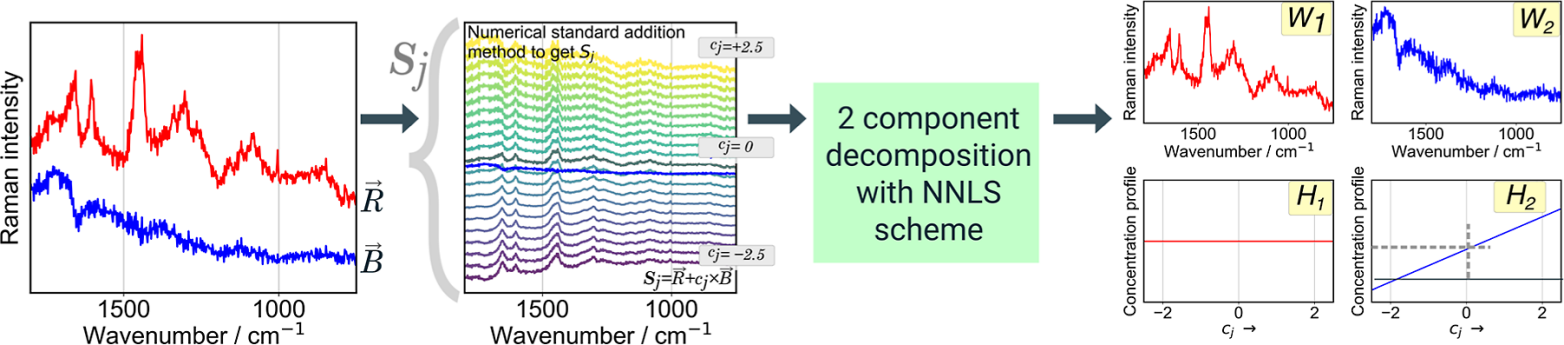
Visual description of the determination of the subtraction coefficient for a known spectral vector B⃗ in a mixed spectrum R⃗ using HAMAND. The scheme is based on using numerical standard addition method followed by 2-component Non-Negative Least Squares (NNLS) decomposition. The shown spectral range and values of coefficient *c*_*j*_ are for illustration.

When processing the present data set using HAMAND, the unknown spectrum was the denoised Raman spectrum ( ${\mathrm{R⃗}}_{i}$ ), while the standard was the background spectrum ( B⃗ ) and the coefficient, *c*_*i*_, was determined for each of the *i* ^th^ spectra by an iterative HAMAND run.

### 2.5 Image and Spectral Cleanup in Processed Data Set

The mapped area during Raman measurements may contain partially overlapping cells, in addition to the cell being studied. It becomes important to distinguish these unwanted regions from the target area to assess the net Raman intensity from a target peak and the associated statistics at the single-cell level.

When the summed band intensities from all mapped points of a cell are compared, further consideration is needed with regard to the cell size. Individual cells exhibit a large variation in cell size, which also changes with stages of the life cycle. Thus, normalization of the net band intensity observed in a map with regard to the cell size becomes necessary when comparing molecular concentrations via Raman intensities among different cells. In the present mapping measurements, the axial resolution is much larger than the cell diameter, implying that vertically the whole cell is measured at once. Hence, a 2D Raman map is constructed to discuss the one cell, and a normalization for the cell’s lateral area is sufficient.

In order to achieve both of the above objectives, selective cleaning of the Raman map (and corresponding Raman spectra) and area determination from the Raman map, a point-wise selection tool was devised. This was implemented using point-wise user-adjustable markers, which formed a polygon on the image map. The area bound by the polygon was immediately available by counting the number of mapped points inside the polygon multiplied by the unit map area (which was 0.16 μm^2^ in the present case).

Further, the polygon region on an image map was indexed to the Raman spectral data set, and thus, all the corresponding spectra inside (or outside) the polygon could be extracted simply by referring to this defined index.

### 2.6 Peak Amplitude Determination in the Processed Data Set

During analysis of the raw and processed Raman data set, the peak parameters were determined through a least-squares curve fit assuming a line shape as a Gaussian function with a linear baseline (see Section SM5 for additional details). Numerical constraints relevant to peak position, bandwidth, and amplitude were provided, and analysis over the full data set was performed via programmed for-loops for rapid processing in an unattended manner.

The above mathematical operations, described under Section 2.2, were implemented as computer code in IgorPro^32^ software for rapid execution of the data analysis process on multiple data sets.

## 3 Results and Discussion

Raman spectra as well as GFP fluorescence spectra in the anti-Stokes region were acquired from four different *S. pombe* cells in independent experiments (see Table 1 and Section 2 for details). These data sets were analyzed according to the scheme illustrated in Figure 2. In the first step, the data set was corrected for irregular sensitivity of the detector by dividing all spectra with the normalized spectrum of broadband white-light emission from a tungsten filament lamp (see Section SM1 for additional details), and wavenumber calibration was performed. Next, the data analysis procedures were sequentially applied: (*i*) denoising using low-rank approximation; (*ii*) automated background determination; (*iii*) background removal using a space-dependent coefficient from HAMAND. Lastly, to evaluate the analysis scheme for quantitative analysis of a single cell, we extract contributions of one cell from the mapped image. The Raman imaging data set from cell 1, comprising of 588 spectra, is discussed in detail below.

**Figure 2:**
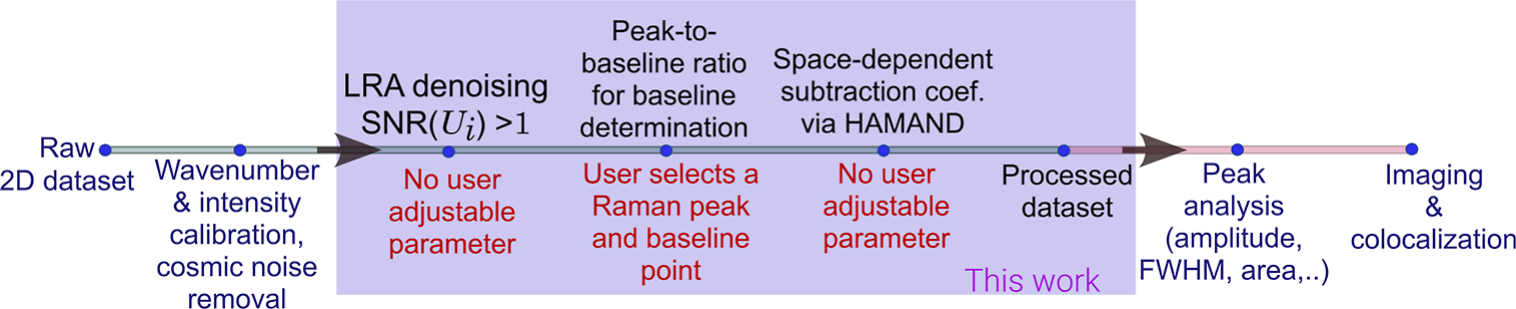
Scheme of analysis employed in this work. New techniques introduced are shown in blue color. See Section 2.2 for further details.

In the first step, the low-rank approximation (LRA)-based denoising procedure was used (as described in Section 2.2.1). In our analysis, the first 15 spectral components were selected out based on the criteria of SNR­(*U*_*i*_) > 1. This criterion retains all spectral information at the cost of noise removal. Results, including the spectral vectors and principal values, are shown in Section SM3 (Figure S11 in the Supporting Information). Only a nominal improvement in the SNR was observed in the Raman mapping image constructed using the 1443 cm^–1^, see Figure S11d,e for a comparison. The corresponding spectral data still contained noise hindering further analysis of Raman data, visualized as the denoised spectrum in Figures 3a and 4a. This is attributed to the inclusion of a large number, 15, of SVD spectral components during the reconstruction of the data set.

**Figure 3:**
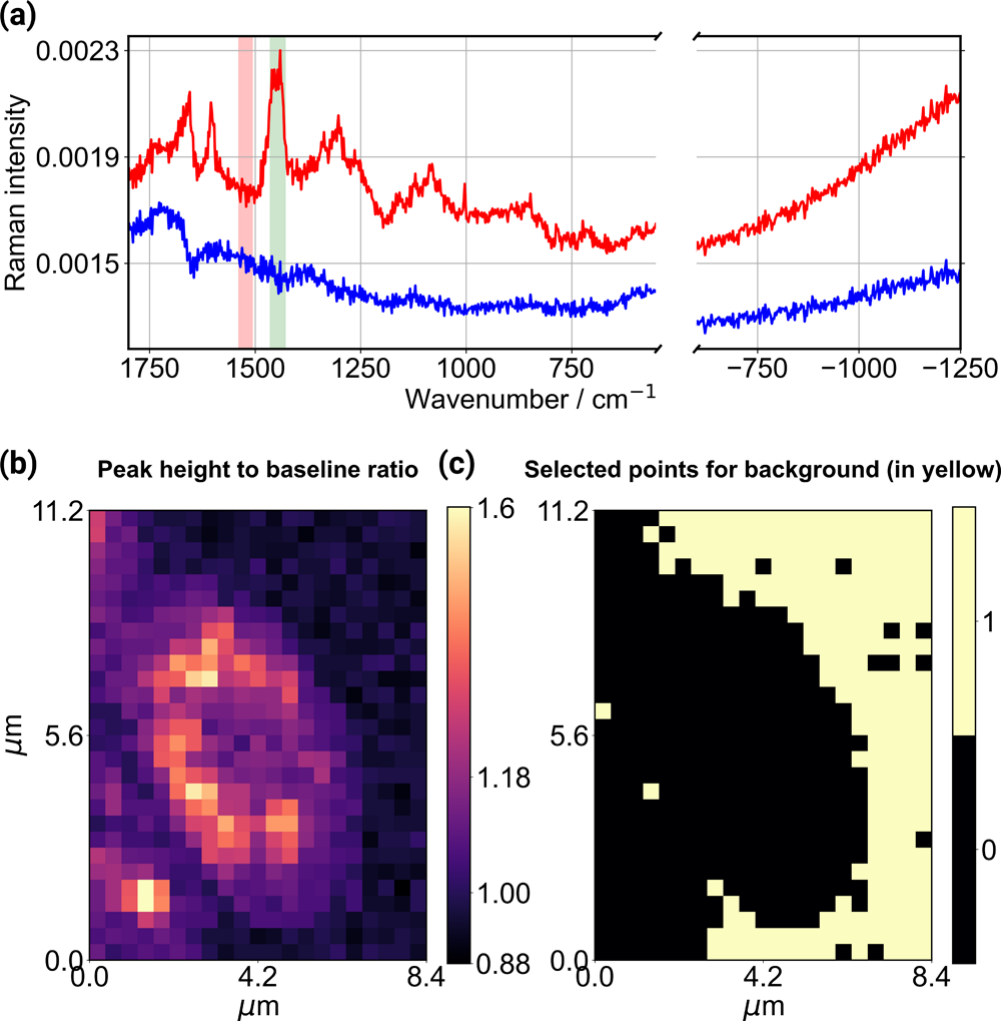
Details of the peak-height-to-baseline filter for automated determination of the background spectra. (a) Representative Raman spectrum from inside the cell (red trace) where the Raman peak used for the filter is indicated in the green shaded region, and the area used for the baseline is indicated in the red shaded region. (b) Peak-to-baseline intensity ratio for all the spatial points in the Raman map. The Raman spectra from points having a ratio ≤1.0 were averaged to determine the background, shown as blue trace in (a). Lastly, in (c), the spatial points identified from the peak-to-baseline intensity ratio ≤1.0 in yellow exemplify that these points are exclusively from outside the cell.

**Figure 4:**
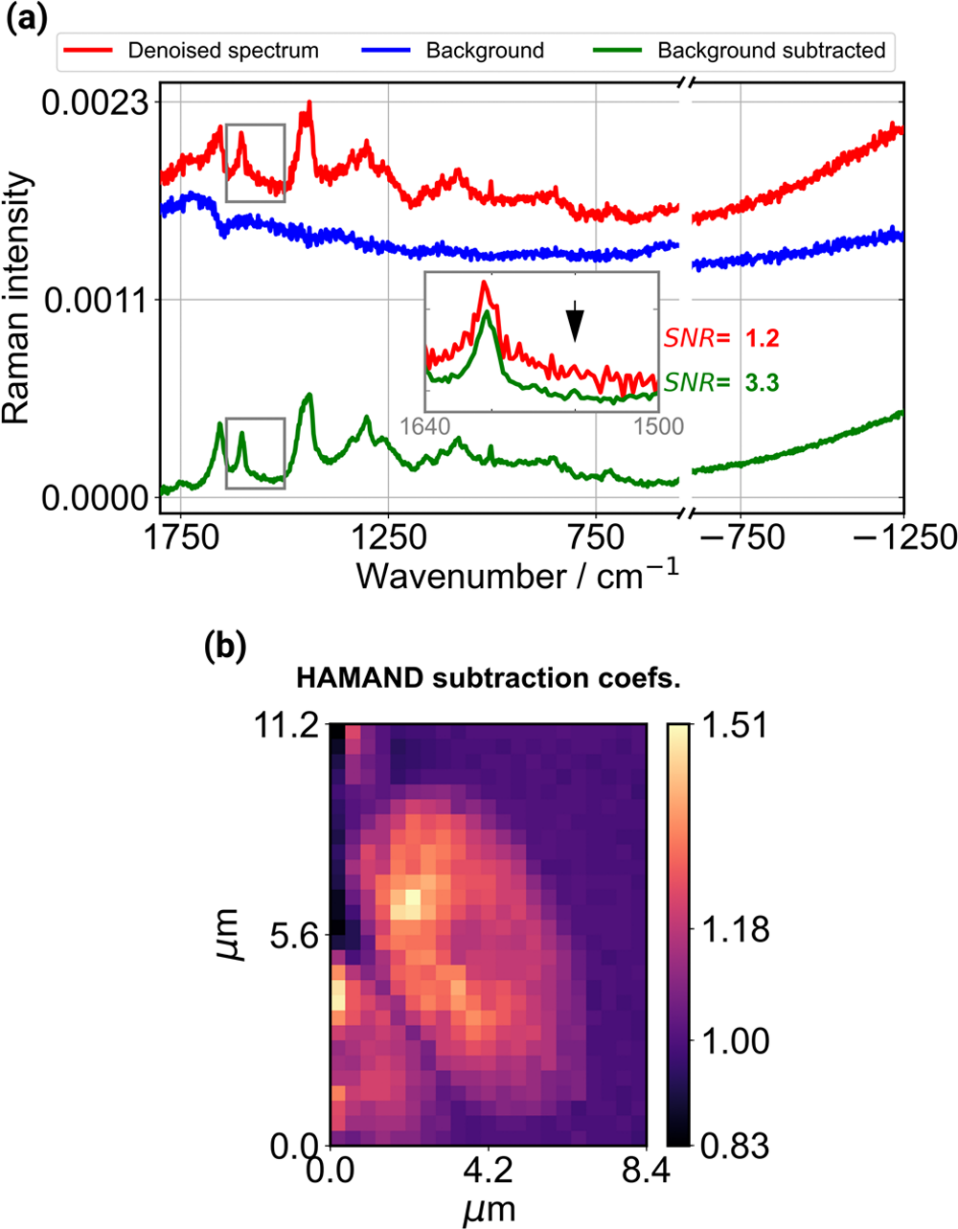
(a) Raman spectra from a point inside the *S. pombe* cell illustrating the effect of the data analysis process. The spectrum after low-rank approximation-based denoising is shown in red with an offset. The background spectra, determined automatically, are in blue, and last, the final spectra after subtracting the background using HAMAND are in green (the automatically determined coefficient was 1.085 for this specific spectrum). The inset shows the zoomed-up region between 1500 and 1640 cm^–1^ of the original and background-subtracted spectra, respectively. The 1550 cm^–1^ peak indicated with an arrow is discernible from noise only after subtraction of the background. (b) Spatial visualization of the subtraction coefficient obtained from HAMAND.

Next, the results of the baseline determination from the Raman data set are summarized in Figure 3. Sub-Figure 3a shows one representative spectrum of the Raman data set from inside the yeast cell. The Raman peak used for the analysis is indicated in green color (which corresponds to the Raman signal from the CH_2_-bend and the CH_3_ degenerate deformation of the hydrocarbon chains, originating from lipids and proteins), while the region of the spectra used for baseline determination is shown in the red shaded region. Thus, we use a Raman signal specific to the cell to obtain the peak intensity-to-baseline ratio, *I*_*x*,*y*_(1443 cm^–1^), which is visualized in Figure 3b. For the present yeast cell, 228 spectra out of the total 588 were identified from outside the cell when using the filter of ≤1.0 for the intensity ratio. These points are exclusively from outside the yeast cell, as shown in Figure 3c using yellow points. Next, the Raman spectra from these locations outside the cell were then averaged, shown as a blue trace in Figure 3a.

The baseline determination process provided us with an averaged background Raman spectrum obtained from outside the cell. This procedure can be used with any cell-specific Raman peak to identify spectra from inside and outside the cell. We tested the ring-breathing mode of phenylalanine at 1003 cm^–1^ as an alternative choice to find that it yielded similar results (see Section SM4 for more details). A comparison of the background spectra obtained from 1443 and 1003 cm^–1^ peaks shows that these are identical to within 0.05% in the Raman intensities.

Next, the advantage of the above-mentioned procedure is fully realized when the background spectrum is removed from the data set. Figure 4 shows the Raman spectra from one point inside the cell, demonstrating how it changes during the analysis process. The denoised spectrum is indicated in red, and the automatically determined background is in blue. The HAMAND process (see Section 2.4) provided the subtraction coefficient (which was over 1 in this case). Lastly, after subtraction of the background, the final spectrum in green was obtained. The most striking feature is the complete removal of structured noise from the spectrum, which allowed the observation of minor undiscovered features. In Figure 4a, this improvement is exemplified using the 1550 cm^–1^ peak. This small feature was indistinguishable from noise before subtraction of the background. The SNR of this peak was computed using peak amplitude as the signal and noise from the 1σ standard deviation in the spectral region having no Raman features spanning from 1500 to 1530 cm^–1^. Prior to background removal, SNR was 1.2, which improved to 3.3 after subtraction. The peak is now easily identified, and the removal of noise enabled us to construct the Raman map of this weak signal (as shown in Figure 7 and discussed later in the text).

The refractive index of a cell is typically *n* > 1.34 depending on the location in the cell and specific concentration of biomolecules therein.^33^ Lipid droplets show a refractive index as high as 1.42.^34^ This is higher than the refractive index of the cell growth medium prepared in water (*n* = 1.33 to 1.34). Considering Raman measurement in the backscattering geometry, with a higher refractive index of the cell, the focal point shifts toward the upper *z*-axis due to refraction at the glass–sample interface. This results in the Raman signal originating from the growth medium to be detected with varying contributions at different mapping points (visualized in Figure 5, see Section SM9 for a quantitative estimate). Removal of the background spectrum should account for this non-unity contribution and necessitates the determination of a space-dependent (or location-specific) subtraction coefficient. For this purpose, we used HAMAND.

**Figure 5:**
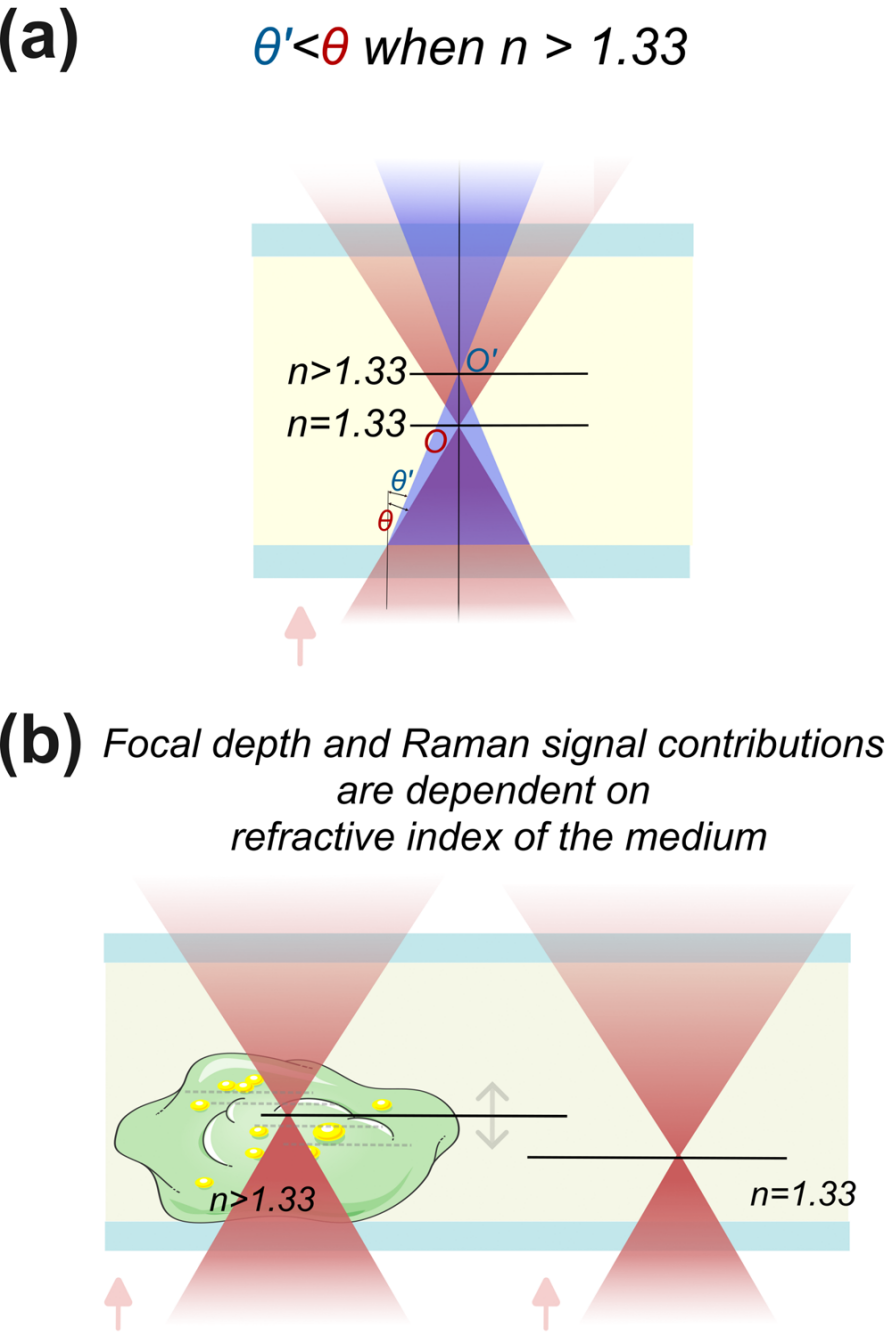
Illustration showing the shift of the focal spot, with variation of the refractive index of the medium. Laser propagation direction is from bottom to top. (a) Refraction at the coverslip–medium interface results in a shift of the geometrical focal point from *O* to *O*′ when the refractive index changes from 1.33 (water) to a higher value. (b) Analogous illustration where a laser is focused to measure the optical signal from a cell (typically *n* > 1.34), and thus, a minutely different depth of focal point at different spatial positions is expected.

The subtraction coefficients determined from HAMAND are visualized in Figure 4b showing that they vary significantly depending on the measured location. For points outside the cell, the values are close to one. However, inside the cell, this coefficient gradually changes over unity. This space-dependent change and increase in the contribution of the background from inside the cell are attributed to focal point shift. In addition, the contribution of fluorescence due to laser excitation from the cell cannot be ruled out. We found that the background level changed inside the cell to a large extent only by a multiplicative factor of the obtained background in the presently analyzed cells. However, additional fluorescence, for example, large cellular autofluorescence, can cause degradation of background removal performance (see Discussion on limitations).

Continuing the analysis, the obtained HAMAND coefficients were used to remove the background from each spectrum of the Raman data set. The resulting spectra had no negative features, and minor Raman peaks were now noticeable, as already shown in Figure 4a. In the usual approach, i.e., without a space-dependent determination of the subtraction coefficient, one may expect many spectra from inside the cell to be contaminated with remaining background or may suffer from oversubtraction resulting in negative features.

Another important aspect is the presence of the GFP signal in the anti-Stokes region (Figure 4), which gives useful information about the localization of a specific organelle. This spectral region resembles a continuous baseline and has no Raman features, thus hindering the applicability of typical baseline removal techniques that rely on peak identification. The HAMAND subtraction of automatically determined background is free from such restriction and thus allows us to reliably extract the features of the fluorescent label.

Figure 6 shows the monovariate Raman images constructed using the Raman peak at 1443 cm^–1^ from different stages of postprocessing. The Raman image after the denoising process is shown in Figure 6a, which has an offset in the Raman intensity due to the background. After subtraction of the background using HAMAND, the resulting image in Figure 6b shows a markedly reduced noise. A large improvement in the image contrast in subcellular regions due to the reduction of the background level is observed. Lastly, for discussing quantitative chemical information via Raman intensities on a *per-cell* basis, we use a mask-based procedure to clean up the portion of extra cells in the Raman image in Figure 6c. For this masking purpose, a polygon bounding the yeast cell was constructed using 9 user-supplied 9 points on the Raman map. The Raman spectra corresponding to the rejected region were all set to zero in the cleaned Raman data set. This final data set, corresponding to a single cell, is then ready for a quantitative study of cellular chemistry focusing on individual molecules (via Raman peaks) or broader comparison with other cells by analyzing their analogous data sets.

**Figure 6:**
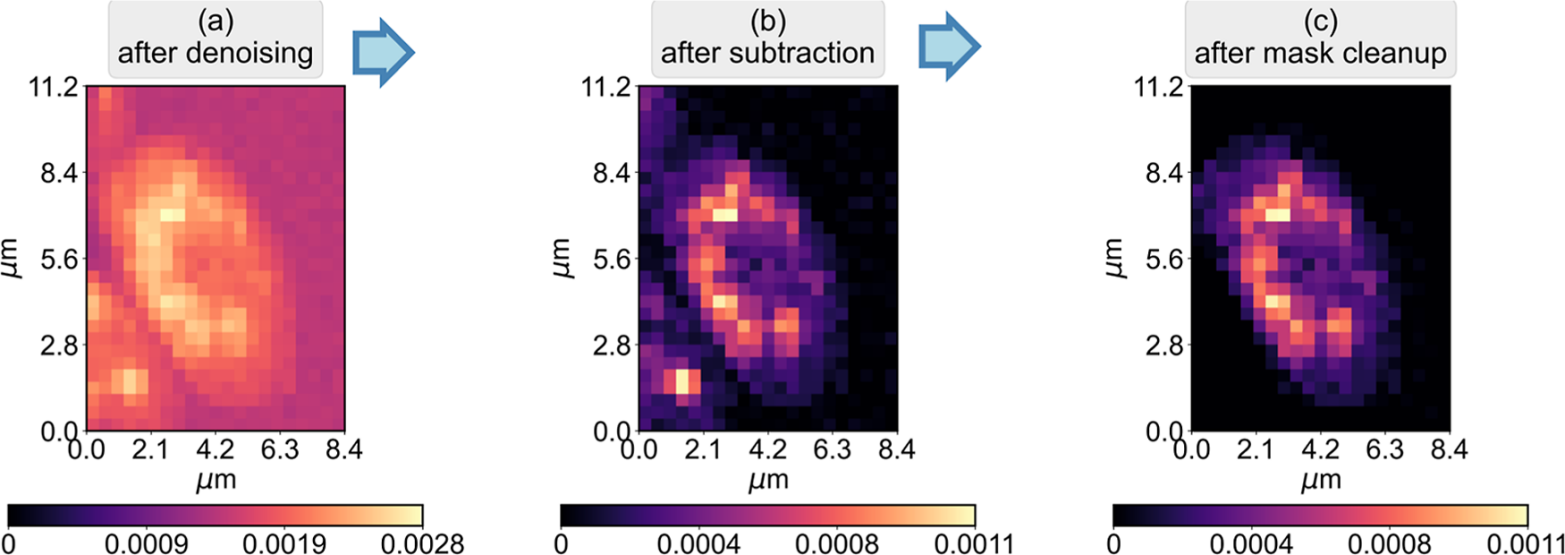
Summary of the data analysis presented in this work. (a) Monovariate Raman image constructed using the Raman peak at 1443 cm^–1^ from the denoised data set, (b) image constructed after subtraction of the automatically determined background, and (c) analogous image after spectral cleanup to remove portions of cells using the mask-based procedure.

### 3.1 Identification of Minor Peaks

How does the present data treatment translate to Raman mapping images and chemical analysis? To answer this question, Raman maps were constructed using the peak amplitudes obtained from the least-squares curve fit from the processed data set. The Raman maps showing the amplitudes of 4 vibrational signatures: 1003 (ring-breathing of the phenyl-group in phenylalanine), 1550 cm^–1^, 1602 cm^–1^ from ergosterol localized in lipids^35^ and 1745 cm^–1^ from the carbonyl stretch of the ester linkage of phospholipid^36^ are shown in Figure 7a–f. The spatial correlation between the maps over all pixels was analyzed using the Pearson correlation coefficient, with a value close to 1 representing high correlation and colocalization, while −1 represents a negative correlation.^37^ This information is visualized in Figure 7g.

**Figure 7:**
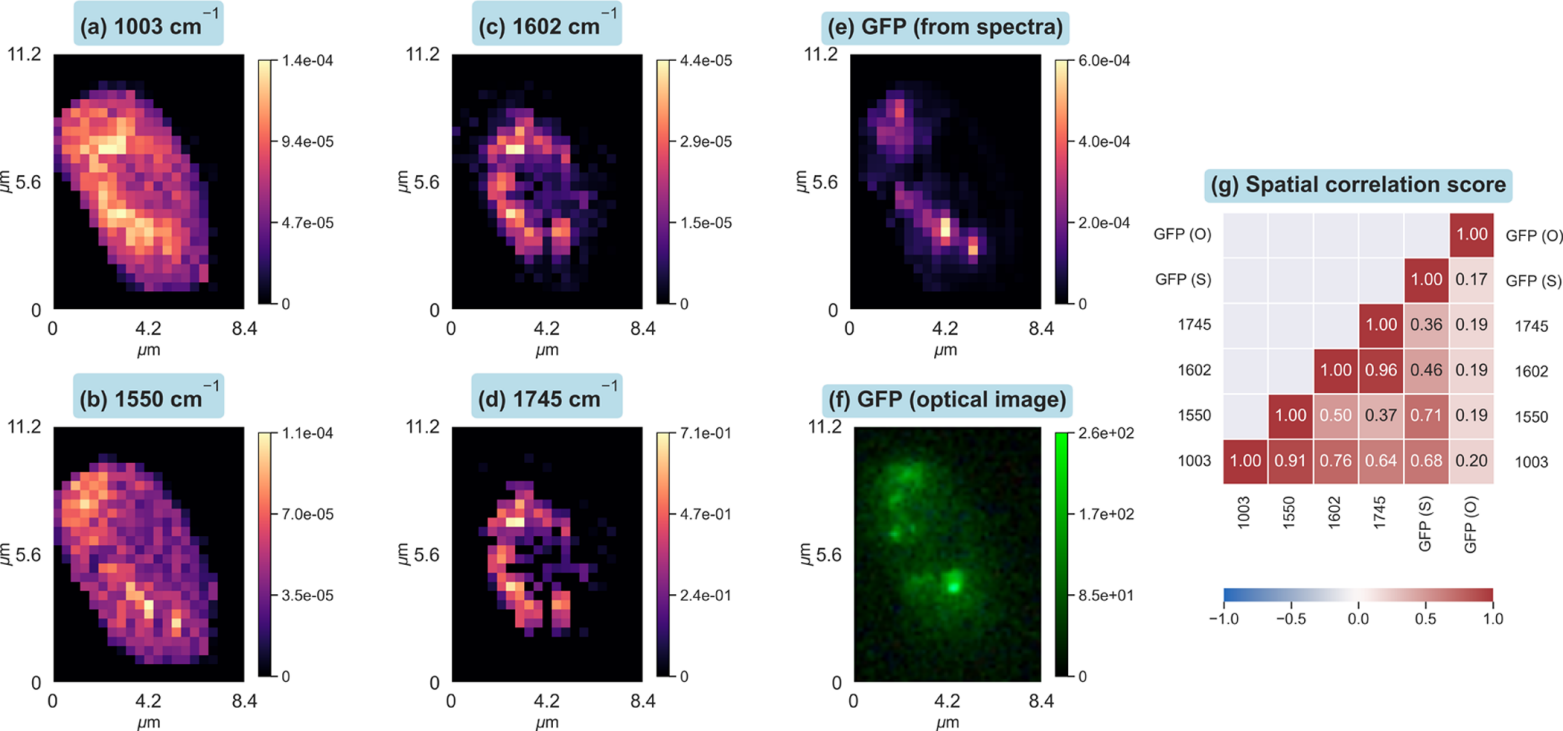
Monovariate Raman images of four Raman bands in (a–d) constructed using the Raman peak at 1003 cm^–1^ from phenylalanine, 1550 cm^–1^ from tryptophan, 1602 cm^–1^ from ergosterol, and 1745 cm^–1^ peak from ester carbonyl stretch. Image of GFP constructed from the intensity on the anti-Stokes region of the Raman spectra in (e) and the optical image of GFP captured prior to the Raman acquisition in (f). Spatial correlation between the six images is shown in (g) computed using Pearson’s cross-correlation scheme. Peak fitting was used to determine the band area for the discussed Raman bands, and integration was used for the GFP signal (e).

The high spatial correlation between the Raman images of 1003 cm^–1^ and of 1550 cm^–1^ indicates colocalization, implying that the peak at 1550 originates from protein, and we assign it to the W3 mode of tryptophan.^38^ The ester carbonyl stretch of phospholipid at 1745 cm^–1^, which is usually too weak to be analyzed precisely, is successfully mapped, showing a high correlation with 1602 cm^–1^ originating from ergosterol. These molecules have been shown to be relatively strong in highly convoluted inner membranes of mitochondria in actively growing and dividing cells.^19^ In addition to the peak amplitudes, peak bandwidth and position were obtained with high accuracy, which opens new ways to discuss minor Raman peaks, for example, protein conformation via the amide-I band.^39^

GFP intensities were obtained from two measurements: one from the optical image captured prior to the Raman measurement, marked as GFP­(O), and the second from GFP intensity extracted from the anti-Stokes region of the processed Raman data set, marked as GFP­(S). The protein signals (1003 and 1550 cm^–1^) show high correlation with the GFP­(S) image, both measured in the same Raman acquisition. This demonstrates a parallel Raman-GFP mapping. Successful retrieval of the GFP map from the Raman data set, as shown in Figure 7e, would enable the study of mitochondrial-tag and location-specific Raman features via image correlation, which is aimed to be performed in the future.

### 3.2 Tests of Reproducibility

The robustness of our analysis scheme was assessed by studying four data sets having different noise content. The quality of these data sets was measured by computing the SNR of the most prominent, 1443 cm^–1^ peak of the averaged top 20 most intense Raman spectra in each data set. SNR was computed as (peak height/noise), where height was obtained as the difference of mean intensity of three consecutive points centered at 1443 cm^–1^ to the intensity at the flat region centered at 1510 cm^–1^; and noise was the 1σ std. deviation in the region from 1510 to 1525 cm^–1^. This measure of data quality indicates the upper level of SNR of the 2D data set.

The determined SNR ranged from 36.5 (for C1) to 5.4 (for C4) in the raw data set. After processing, this improved, reaching 81 to 22, respectively (visualized in Figure 8). We then focus at the weak 1550 cm^–1^ peak, which is otherwise unidentifiable in the raw data set in all the analyzed data sets. Peak amplitude was obtained after performing a constrained fit in the wavenumber region of [1530, 1575] cm^–1^ for both of the data sets, respectively. Results are summarized in Figure 8 using monovariate images. In the unprocessed data set, the peak-fit either fails or yields unreasonable fit parameters at most spatial points. The cellular boundary is unrecognizable. After data processing, amplitudes for the weak 1550 cm^–1^ peak were successfully obtained in these four different data sets with clear demarcation of the cell. This shows the applicability of the present analysis scheme to data sets of varying quality and types.

**Figure 8:**
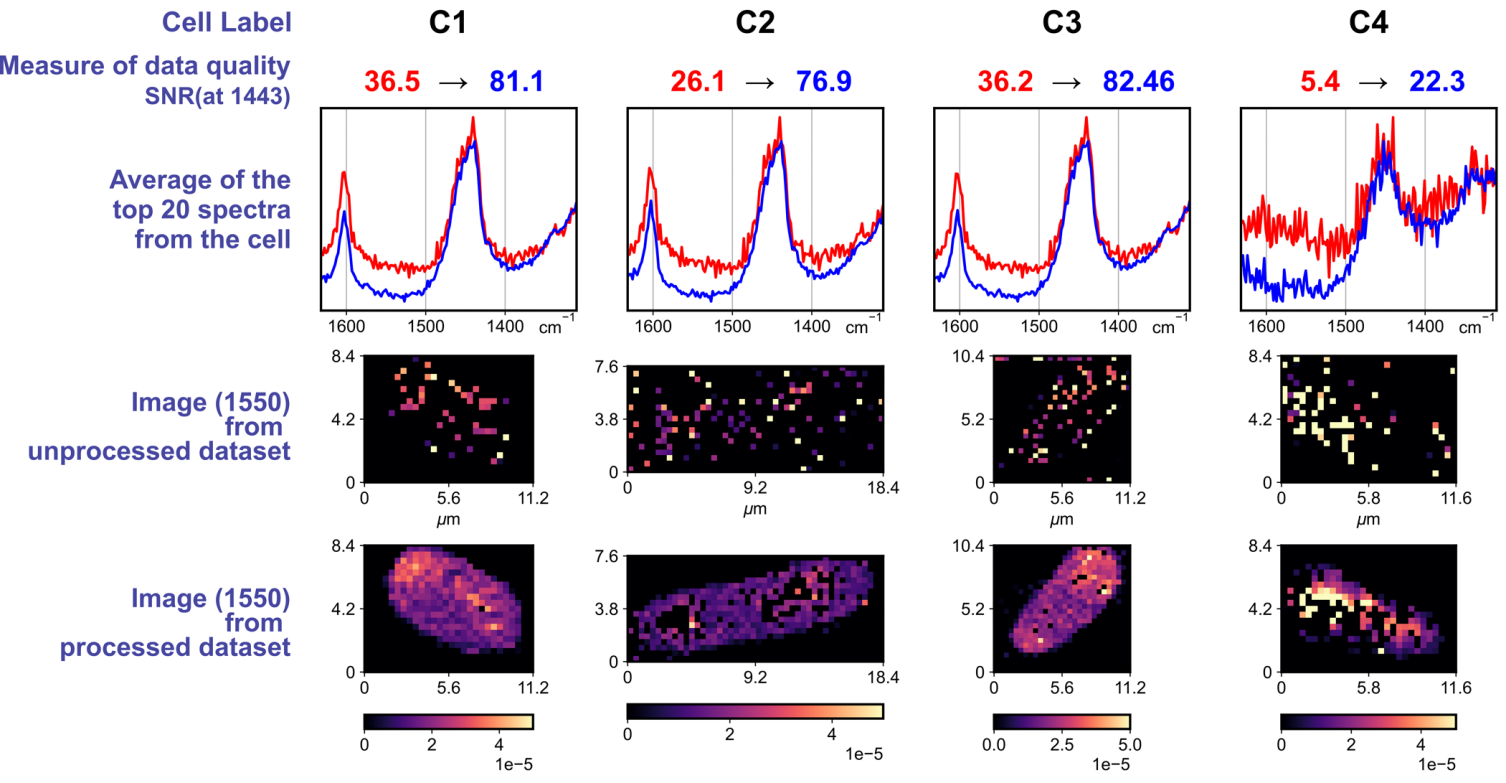
Performance evaluation of the present analysis workflow with four data sets of varying quality. The data quality was assessed by looking at the SNR of the 1443 cm^–1^ peak in the averaged spectrum of the most intense top 20 spectra (see text for details). SNR from the raw data set is in red, while that of the processed data set is in blue. The corresponding average of the top 20 most intense spectra is shown in the same colors. The peak amplitude from the least-squares curve fit for the 1550 cm^–1^ peak was used to prepare the monovariate images (bottom two rows) showing clear identification of this minor peak after postprocessing.

### 3.3 Comparison with “Baseline Estimation” Methods

Background determination and careful removal are the core aspects of this work. It demands a detailed discussion, particularly of how it compares to other techniques. A number of algorithms have been proposed aiming at the estimation of the baseline via different mathematical schemes. Among the most widely used ones are those based on asymmetric least-squares (ASLS)^40,41^ and derivative methods,^42,43^ morphological penalized least-squares (MPLS)^44^ and subalgorithms,^45,46^ spline-based methods,^47,48^ and iterative techniques based on smoothing.^49^ These schemes aim at determining a smooth baseline connecting all or most spectral points where no Raman features are present, evaluated typically within a least-squares optimization framework. Thus, the determined baselines are purely mathematical constructs. In stark contrast, the background automatically determined in this work is the Raman signal of the substrate in combination with that of the growth medium, which therefore has a clear physical basis for its shape.

Consequently, a direct comparison of our method to those of other techniques of baseline estimation should be done with caution. For completeness, results from one such analysis are presented below in Figure 9. The baseline estimated from ASLS, ARPLS, MPLS, and masked polynomial fit with orders 4 and 5 was subtracted for one of the Raman spectra in the data set. These mathematical methods, however, require parameters which govern the target spectral region, optimization gradient tolerance, and/or smoothness, details of which are placed in Section SM6 of the Supporting Information. Results from the present work, where the background Raman spectrum was determined and removed automatically, are shown for comparison. In Figure 9, minor peaks, such as those at 1550 and 1745 cm^–1^ are clearly visible only in the present result. The spectral profiles obtained after removal of baselines determined from all mathematical algorithms are artificially flat, and the line shapes are obscured, for example, peaks at 1655 and 1443 cm^–1^. The line shape of the 1443 cm^–1^ band is well characterized for lipids.^50^ This peak is asymmetric with the peak-top toward the lower wavenumber side, typically ranging from 1440 to 1443 cm^–1^ (for example, see Figure 8 in ref 50). This line shape is correctly obtained only in the present approach, indicating that the background estimation and removal was indeed correctly performed. The improvement in the SNR is most remarkable. This performance results from the fact that we are subtracting the Raman profile of (substrate + growth medium) from the raw spectrum with a proper coefficient.

**Figure 9:**
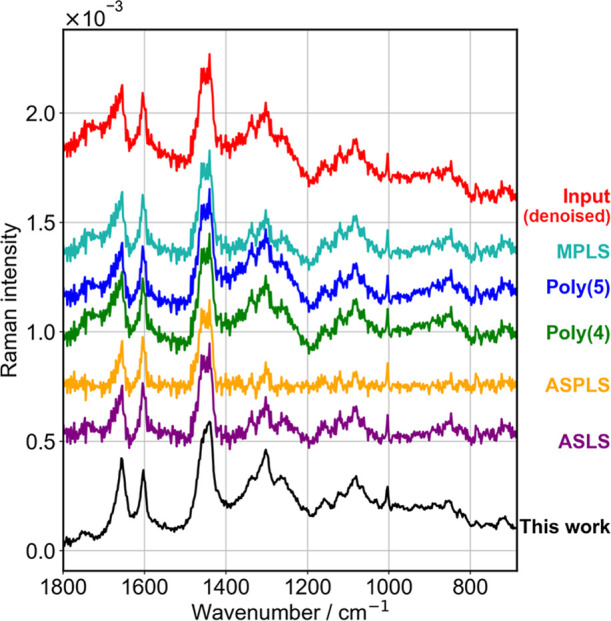
Comparative evaluation of the Raman background removal via HAMAND in this work, with baseline estimation from 5 methods and subtraction using factor of 1. Input spectrum (denoised) is in red, while baseline-subtracted outputs are shown in other colors. Spectra are shown with an offset to aid in visualization.

Lastly, in principle, one can remove systematic noise from the spectra after removal of a smooth baseline by extraction of noise features and least-squares subtraction. We performed this in one step by using the HAMAND subtraction coefficient.

Proceeding further, when the resulting background-subtracted data sets from the above-discussed techniques are used for image construction, the improvement to Raman images delivered by our method becomes clearer. Figure 10 shows the Raman images for the 1550 cm^–1^ peak as a comparison. Standard workflows based on smooth baseline estimation result in a Raman data set where minor peaks cannot be accurately analyzed. This is caused by the failure of the least-squares fit at about 50% of points inside the cell and/or a large error in the Raman amplitude. In contrast, the present workflow delivers a Raman data set which yields reliable peak amplitudes from the fit at all spatial points in the cell, with reasonably small errors.

**Figure 10:**
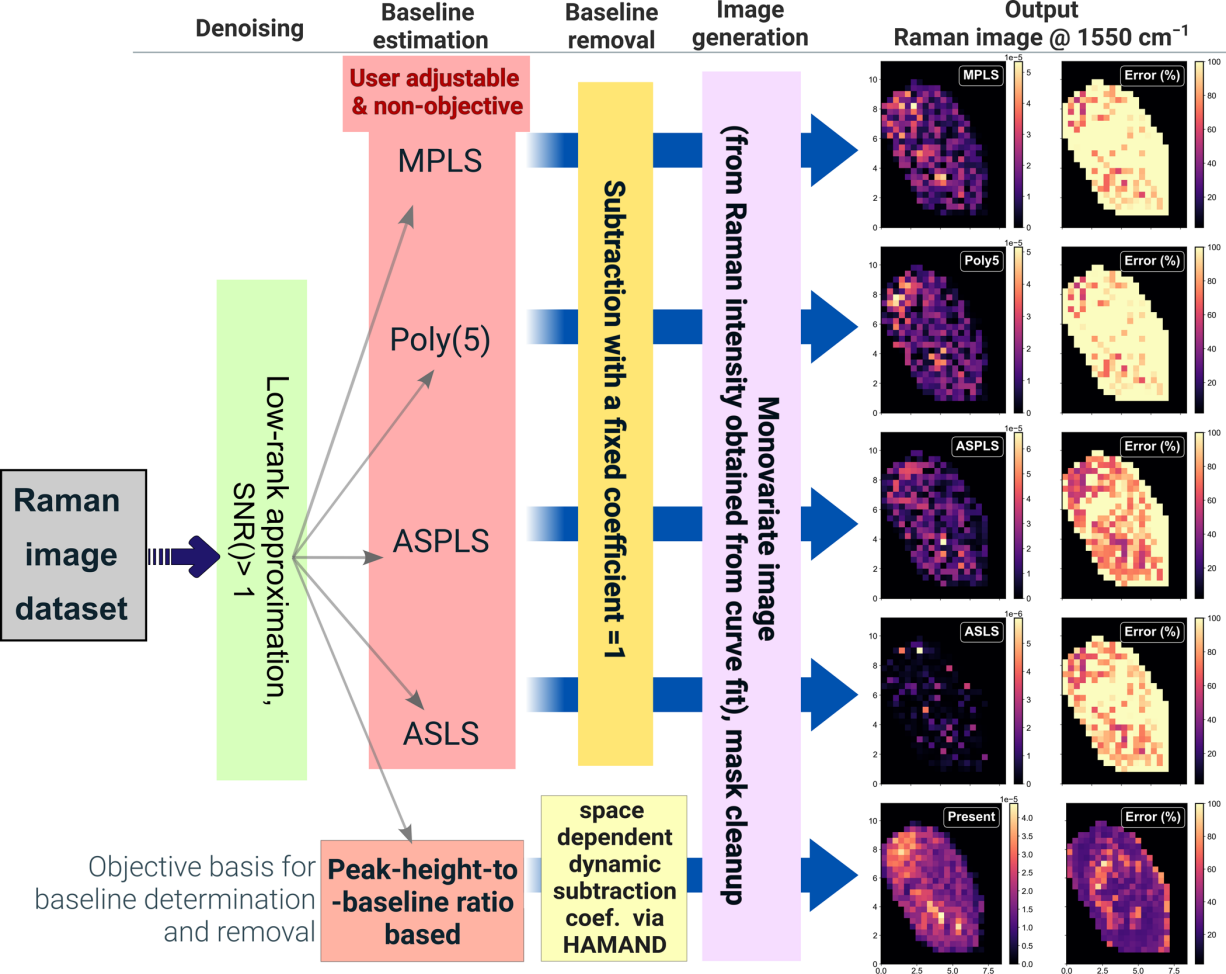
Raman image output for the 1550 cm ^–1^ peak obtained from various analysis workflows. Four techniques for baseline and removal are compared with the present technique. The present approach, based on automated background determination and its HAMAND removal, delivers a processed data set which can be analyzed accurately for Raman intensities for all points in the cell, providing a Raman map with less uncertainty in the intensity.

Apart from the above-discussed methods, another approach to analyze large spectral data sets is non-negative matrix factorization (such as MCR-ALS), which, however, requires the number of components to be known or guessed. MCR-ALS was performed for the present data set (C1) assuming 9 spectral components, as indicated by SVD analysis (see Figure S11). The results showed that the obtained spectral vectors were severely mixed, with many of them having negative features. Furthermore, even if the background spectrum was provided as one of the fixed components, the performance of MCR-ALS did not improve (see Section SM7 for the full set of results). This indicates the limitation of MCR-ALS when the number of spectral components is not known beforehand.

### 3.4 Advantages and Limitations of Present Workflow

The distinction of the present method, background determination by analyzing 2D data set and HAMAND removal, with that of baseline “estimation” through a mathematical approach and subtraction is thus made clear. The advantages of the present method are multifold: (*i*) the removal of the determined background does not obscure line shapes of the remaining Raman bands (Figure 9), (*ii*) a variable subtraction coefficient (determined by HAMAND) effectively accounts for a changing contribution of background from growth medium in the confocal volume (Figures 4 and 5), (*iii*) removal of the determined background, which will have noise, effectively eliminates structured noise present in the data set (Figures 4 and 8); and last, (*iv*) the approach works across the spectral range provided that one signature peak is available somewhere for the peak-height-to-baseline filter (thus allowing us to analyze the anti-Stokes region unambiguously). Another benefit is that only a small set of physically meaningful parameters (positions for Raman peak and baseline) are required for background determination.

A major limitation of the proposed analysis scheme is dealing with strong autofluorescence, which can affect the analysis workflow in distinct ways: (*i*) If the spectral shape of autofluorescence is nonchanging, its model spectra can be isolated from the data set and selectively removed. For such a use case, another iteration of HAMAND subtraction (for eliminating pure autofluorescence spectrum) should be performed. This approach would be useful for lower wavelength Raman laser excitations, such as 532 nm, where higher autofluorescence is expected. (*ii*) However, if the spectral shape of autofluorescence changes spatially, a reliable separation of this fluorescence signal becomes prohibitively difficult, and further investigation is required to analyze such cases. (*iii*) Lastly, if the autofluorescence is so large that all Raman signal are completely overshadowed, and a unique Raman signal, such as 1443 cm^–1^, specific to the cell, is unidentifiable, the introduced background determination scheme becomes unusable.

## 4 Conclusion

In this work, an objective scheme for spectral postprocessing directed toward Raman molecular mapping analysis of single cells is presented. The multistep procedure involves denoising, objective background determination, and its HAMAND subtraction, followed by usual Raman spectral analyses. This process is demonstrated using Raman mapping data sets of *S. pombe* cells. We were able to identify otherwise unidentifiable minor peaks and construct their Raman maps to discuss distribution inside the studied cells. Robustness was assessed by analyzing data sets with different SNR. The proposed scheme is readily programmable and suitable for rapid analysis of large spectral data sets. It constitutes an important step toward quantitative analysis of molecular abundance and distribution in living cells.

## Supplementary Material



## Notes

Additional details on Raman measurements, tests on the low-rank approximation for noise reduction in spectral data set, results on low-rank approximation for data on Cell 1, baseline determination using the protein peak at 1003 cm^–1^, results on curve-fit for peak amplitude determination, comparison of results with other algorithms (including details relevant to methods discussed in Figure 9), result of MCR-ALS decompositions, results from three other cells of *S. pombe*, and estimation of focal-point change with refractive index of the medium (PDF)
