# Supplementary material for: Posthandling Spectral Information Enhancement for Single Cell Raman Molecular Mapping Analysis
Source: Anal Chem. 2025 Oct 27;97(45):25067–77. doi: 10.1021/acs.analchem.5c03915 (PMC12631732; doi:10.1021/acs.analchem.5c03915)
Supplement: Supplementary file 1 [file ac5c03915_si_001.pdf]

SUPPORTING INFORMATION

Post-handling spectral information enhancement for single cell Raman molecular mapping analysis

Ankit Raj[a,b*], Nungnit Wattanavichean[a,c], Makoto Kawamukai[d], Tatsuyuki Yamamoto[d], and Hiro-o Hamaguchi[a*]

a. Department of Chemistry and Institute of Molecular Science, National Yang Ming Chiao Tung University, 1001 University Road, Hsinchu, 300, Taiwan

b. Department of Chemistry, Faculty of Science Gakushuin University, 1-5-1 Mejiro, Toshima City Tokyo, Japan 171-8588

c. School of Materials Science and Innovation, Faculty of Science, Mahidol University, Phuttamonthon 4 Rd., Salaya, Nakhon Pathom, 73170, Thailand

d. Department of Life Sciences, Shimane University, 1060 Nishikawatsucho, Matsue, Shimane, 690-8504, Japan

[* ankit.sciwork@gmail.com , hamaguchi@nycu.edu.tw]

Contents

List of Figures 2

SM1. Details of the Raman Spectrometer and measurement 3
A. Additional details on measurements 5

SM2. Tests on the low-rank approximation (LRA) for noise-reduction in spectral-dataset 5
A. Definitions 5
B. Analysis on artificial dataset 6
1. Noise magnitude sDev = 15 7
2. Noise magnitude sDev = 30 9
3. Summary 10

SM3. Results on the low-rank approximation based denoising of Raman dataset : Cell 1 11

SM4. Automated baseline determination via protein (signal from phenylalanine at 1003 cm^{-1}) 12

SM5. Results on least squares curve-fitting for Raman peak amplitude determination for image construction 13

SM6. Comparison of results with other methods of baseline estimation 14
A. Details of baseline estimation methods discussed in main document (in Fig. 9) 14
B. Additional comparison 15

SM7. Result of MCR-ALS decomposition 17

SM8. Results from additional *S. pombe* cells 19
A. Cell 2 19
B. Cell 3 22
C. Cell 4 23

SM9. Focal point change with refractive index of the medium 23

Bibliography 25

List of Figures

S1 Layout of the Raman micro-spectrometer used in the present work. 4

S2 Broadband white-light emission from microscope lamp after normalization to the maximum value. 4

S3 Description of the artificial data used for testing. True signals, each having two Gaussian peaks, (in top). Linear baseline shown in bottom. 6

S5 Column 0 of the true and test dataset. For test dataset, the noise level was 15 (centered at zero). 7

S6 Plots showing the deviation in the values of peak parameters (arranged horizontally), obtained for the 6 peaks (shown vertically) as a function of the LRA-rank. The standard deviation of the added noise was 15. Cutoff for LRA approximation obtained from SNR > 1 for U_i is shown as dashed red vertical line. 8

S7 Concentration of original signals 1–3 (or their mixing ratio) in the noisy dataset (sDev = 15) after LRA approximation. Cutoff for LRA approximation obtained from SNR > 1 for U_i is shown as dashed red vertical line. 8

S8 Column 0 of the true and test dataset. For test dataset, noise level was 15. 9

S9 Plots showing the deviation in the values of peak parameters (arranged horizontally), obtained for the 6 peaks (shown vertically) as a function of the LRA-rank. The standard deviation of the added noise was 30. Cutoff for LRA approximation obtained from SNR > 1 for U_i is shown as dashed red vertical line. 9

S10 Concentration of original signals 1–3 (or their mixing ratio) in the noisy dataset (sDev = 15) after LRA approximation. Cutoff for LRA approximation obtained from SNR > 1 for U_i is shown as dashed red vertical line. 10

S11 Results of the SVD denoising process. (a) Top 15 resolved spectral vectors (or basis vectors, (U_i) after SVD analysis of the Raman dataset, (b) Singular values of the corresponding spectral vectors, Σ_i, (c) Signal-to-noise ratio of the spectral vectors computed as per Eqn. (1) in main document, (d) mono-variate Raman image constructed using 1443 cm^{-1} band from the raw dataset, and (e) the analogous image constructed from the denoised dataset. 11

S12 Baseline determined using the phenylalanine signal at 1003 cm^{-1} (in red) and the one determined with the CH_2 wagging signal at 1443 cm^{-1} (in blue). Their difference, ($\text{baseline}_{\text{using 1443}} - \text{baseline}_{\text{using 1003}}$) is plotted in grey with expanded y-axis. 12

S13 Peak amplitude determination using curve-fitting for (a) 1003, (b) 1550, (c) 1602 and (d) 1745 cm^{-1} Raman peaks. A linear baseline with a Gaussian function was used together with initial guesses to perform the least-sqaures curve-fit on spectrum of the processed datasets. A representative fit is shown here. 13

S14 Raw Raman spectrum together with the masked region used for fitting in grey color. 14

S15 Comparative evaluation of the Raman background removal via HAMAND in this work, with baseline-estimation from 5 methods (with subtraction using a factor of one). Input spectrum (labeled as 'Raw') is in red. Spectra are shown with an offset to aid in visualization. See details below for the parameters used in this evaluation. 15

S16 Results from MCR-ALS analysis assuming 9 components for the Raman dataset from Cell 1. 17

S17 Results from MCR-ALS analysis assuming 9 components for the Raman dataset from Cell 1. One of the spectral component, the automatically determined background labelled as 9, was fixed in this analysis. 18

S18 Result from SVD analysis (and denoising) of the Raman dataset for cell 2. (a) Top 14 resolved spectral vectors (or basis vectors, U_i) after SVD analysis of the Raman dataset, (b) Singular values of the corresponding spectral vectors, Σ_i, (c) Signal-to-noise ratio of the spectral vectors, see Sec. SM2 for details, (d) mono-variate image constructed using 1443 cm^{-1} band from the raw dataset, and (e) the analogous image from the denoised dataset. 19

S19 Peak-height-to-baseline filter for automated determination of the background spectra for cell 2. Sub-figure (a) shows the peak height-to-baseline intensity ratio the specific spectra at each pixel. For points outside the cell, this ratio is very close to unity. Sub-fig (b) shows the spatial location of the selected spectra from outside the cell in yellow, and the points from inside the cell in black color. Sub-fig (c) shows the averaged background spectrum from the automated analysis in red color, along with averaged background spectrum from manual selection of 10 points from outside the cell (in blue color) and the 1σ standard deviation is indicated. 20

S20 Summary of the analysis for cell 2. (a) Mono-variate Raman image constructed using the Raman peak at 1443 cm^{-1} from raw Raman dataset and analogous image constructed using the denoised dataset in (b), image constructed after subtraction of the automatically determined background in (c), and (d) the analogous image after spectral cleanup to remove portion of cells. 20
S21 (a) Raman spectra from a point inside the *S. pombe* cell-2 illustrating the effect of data analysis process. The spectrum after LRA based denoising is shown in red. The background spectrum, determined automatically, is in blue, and lastly, the final spectra after subtracting the background using HAMAND is in green. The 1550 cm^{-1} peak is discernible from noise only after subtraction of the background. Subtraction coefficient determined by HAMAND is visualized in subplot (b) 21
S22 Peak-height-to-baseline filter for automated determination of the background spectra for cell 3. Sub-figure (a) shows the peak height-to-baseline intensity ratio the specific spectra at each pixel. For points outside the cell, this ratio is very close to unity. Sub-fig (b) shows the spatial location of the selected spectra from outside the cell in yellow, and the points from inside the cell in black. Sub-fig (c) shows the obtained averaged background spectrum. 22
S23 (a) Raman spectra from a point inside cell-3 illustrating the effect of the data analysis process. The spectrum after LRA based denoising is shown in red. The background spectrum, determined automatically, is in blue, and lastly, the final spectra after subtracting the background using HAMAND is in green. The 1550 cm^{-1} peak is discernible from noise only after subtraction of the background. Subtraction coefficient determined by HAMAND is visualized in subplot (b) 22
S24 Peak-height-to-baseline filter for automated determination of the background spectra for cell 4. Sub-figure (a) shows the peak height-to-baseline intensity ratio the specific spectra at each pixel. For points outside the cell, this ratio is very close to unity. Sub-fig (b) shows the spatial location of the selected spectra from outside the cell in yellow, and the points from inside the cell in black. Sub-fig (c) shows the obtained averaged background spectrum. 23
S25 (a) Raman spectra from a point inside cell-4 illustrating the effect of the data analysis process. The spectrum after LRA based denoising is shown in red. The background spectrum, determined automatically, is in blue, and lastly, the final spectra after subtracting the background using HAMAND is in green. The 1550 cm^{-1} peak is discernible from noise only after subtraction of the background. Subtraction coefficients determined by HAMAND are visualized in subplot (b). 23
S26 Back-scattering geometry is used in the present work, where an objective lens is used for excitation and collection. Illustration shows the movement of converging excitation beam inside the sample while undergoing refraction at the interfaces. Propagation of light at the immersion oil-coverslip interface, and the coverslip-liquid (sample) interface is illustrated. . 24
S29 Change in the focal depth modelled using geometrical optics assuming point focus. 25

List of symbols

λ	:	Wavelength	[nm]
Ω	:	Solid angle	[str]
σ	:	Standard deviation	

SM1 Details of the Raman Spectrometer and measurement

Optical layout of the Raman spectrometer used to acquire spectroscopic data in the present work is shown below.

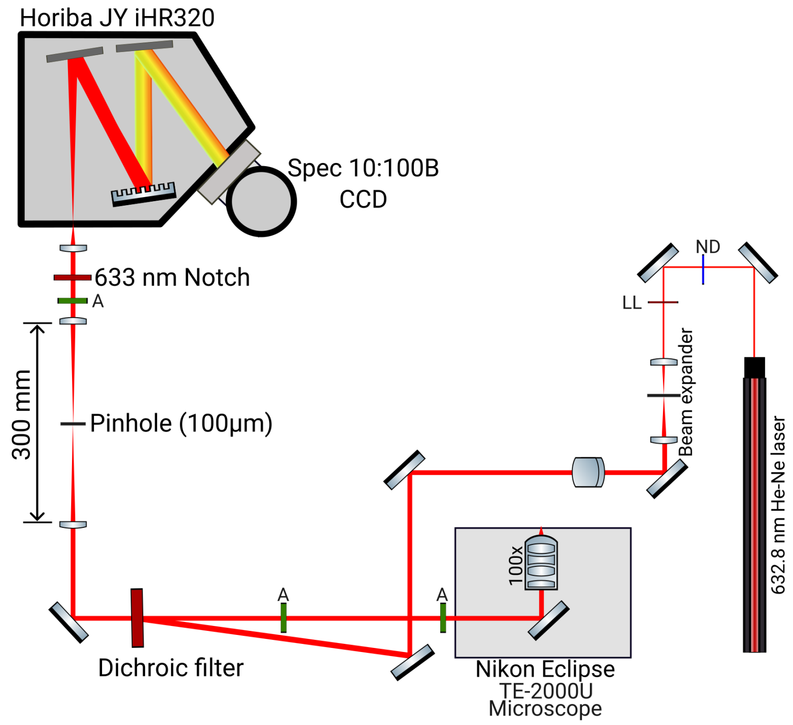

FIG. S1: Layout of the Raman micro-spectrometer used in the present work.

The Raman spectrometer is based on a He-Ne laser at 632.8 nm providing 20mW of output power. In Fig.S1 ND represents neutral-density filter to attenuate the laser. LL represents a laser-line filter, and A represent alignment irises. The laser was directed to the microscope using dichroic filter reflecting 632.8 nm laser at a narrow angle, and allows back-scattered light to pass through towards the polychromator. Rayleigh rejection was done using a notch filter before the entrance slit. Wavenumber calibration was performed using emission lines of neon. Spectral resolution was 1.05 cm^{-1}.

Broadband white-light spectrum acquired from a tungsten-halogen lamp fixed on the microscope was used for relative intensity calibration. The acquired Raman spectra were divided with the normalized white-light spectra. The portion cut by the notch-filter (spanning from 615 to 648 nm, or from -450 to $+390$ cm^{-1} in relative wavenumbers) was manually removed from all the datasets prior to further analysis. Artifacts due to cosmic noise were removed via a custom developed procedure in IgorPro.

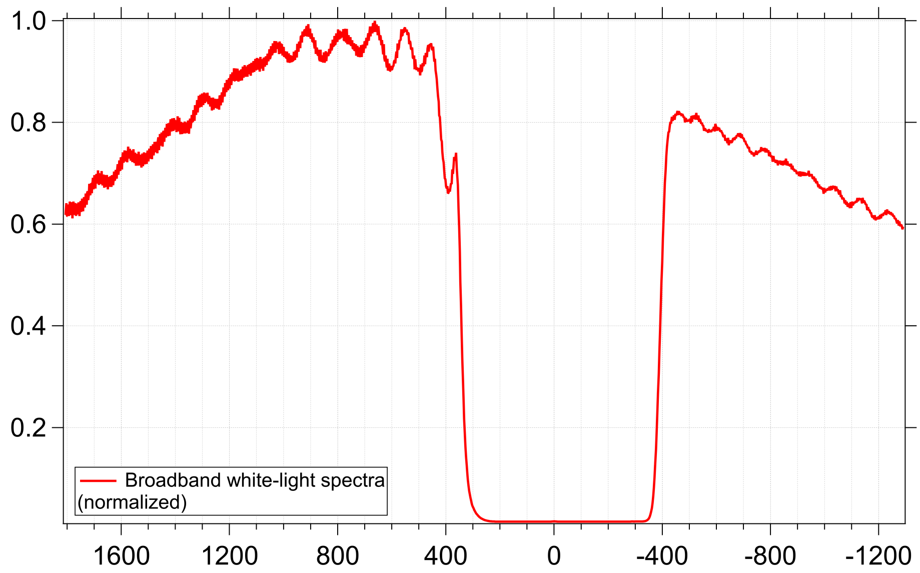

FIG. S2: Broadband white-light emission from microscope lamp after normalization to the maximum value.

A Additional details on measurements

Prior to the spectroscopic measurements, the *S. pombe* cells were cultured in EMM2 cell growth media with 225 mg/L uracil supplements (including 0.5% ammonium chloride, 0.3% potassium hydrogen phthalate, 0.55% sodium phosphate, 2% glucose, salts, vitamins and minerals)[1] at 30°C under aerobic condition, with incubation in a cell shaker operating at 250 rpm. The *S. pombe* cells were cultured for 12 and 24 hours and placed on the poly-D-lysine coated culture dish (P35GC-1.5-14-C, MatTek) and covered with a coverslip to reduce cell movement. All spectroscopic measurements were performed at room temperature (25°C, 50% relative humidity). During the Raman-fluorescence mapping measurements, the *S. pombe* cells were placed on a custom designed holder mounted to a x-y piezo-translation stage (PI E-710.3CD) which was controlled in synchronization with the CCD.

SM2 Tests on the low-rank approximation (LRA) for noise-reduction in spectral-dataset

Noise reduction using the low-rank approximation (LRA) for a $S_{(m\times n)}$ matrix involves reconstructing the original matrix using k singular values where $k << n$.[2, 3] Typically, the largest few singular values are retained and an important consideration is : how many singular values to retain while minimizing information loss and simultaneously achieving maximum noise reduction. To analyze this, the following numerical tests were performed assuming that the original data matrix is S^o, and the reconstructed matrix using k^{th}-order low-rank approximation is $S^{r(k)}$. The dimension of these matrices are $(m \times n)$.

A Definitions

1. SNR(U_i) = $\frac{\sigma(\text{signal})}{\sigma(\text{noise})}$, computed by obtaining the signal by smoothing i^{th} spectral vector, labelled as U_i^s, and the noise is obtained as, noise= $U_i - U_i^s$. The smoothing was performed using the widely-used Savitzky-Golay filter algorithm[4] available in SciPy[5] and IgorPro[6] was used. The degree of smoothing is governed by parameters : `window_length` controlling size of the smoothing window, and `polyorder` controlling the order of the polynomial function used in the smoothing window in a least-squares analysis. In order to reduce the over-dependence of smoothing results on these two parameters, an averaged SNR computed for four different combinations of these parameters was employed (window length varied from 5 to 11 points, and polynomial order varied from 2 to 5, respectively) . See the following code in Python for details:

```

import numpy as np
from scipy.signal import savgol_filter

def compute_snr_eVec(matrix, last_rank):
'''
To compute the SNR of the spectral vectors U_i and return
those in a 2D numpy array (col 5th has the averaged SNR).
Analysis is performed for the i-th U, starting from 1.
'''

    # Perform singular value decomposition (SVD)
    U, S, Vt = np.linalg.svd(matrix, full_matrices=False)

    out = np.zeros((last_rank, 6))

    for i in range(out.shape[0]):
    out[i,0] = i+1
    out[i,1] = compute_SNR( U[:,i] , 5 , 2 )
    out[i,2] = compute_SNR( U[:,i] , 7 , 3 )
    out[i,3] = compute_SNR( U[:,i] , 9 , 4 )
    out[i,4] = compute_SNR( U[:,i] , 11 , 5 )

    # this is the averaged SNR of the ith U vector
    out[i,5] = (out[i,1]+out[i,2]+out[i,3]+out[i,4] ) / 4

    return out

#----------------------------------------------------------------

```

```
def compute_SNR(vector1D, window, pol):
'''
To compute the SNR for a 1D vector using smoothing to get
the signal and noise. 1sigma standard deviation is used.
'''

    # Calculate smoothed values with Savitzky-Golay method
    smoothed = savgol_filter( vector1D, window_length=window, polyorder=pol)

    noise = vector1D - smoothed

    sdev_signal = np.std(smoothed)
    sdev_noise = np.std(noise)

    return (sdev_signal / sdev_noise )

#----------------------------------------------------------------

```

Code block 1: Python implementation showing computation of SNR for spectral vectors

B Analysis on artificial dataset

An artificial dataset was prepared by starting with three true spectral vectors. Each had two Gaussian peaks, thus a total of 6 spectral signatures of varying amplitudes. These are shown in Fig. S3.

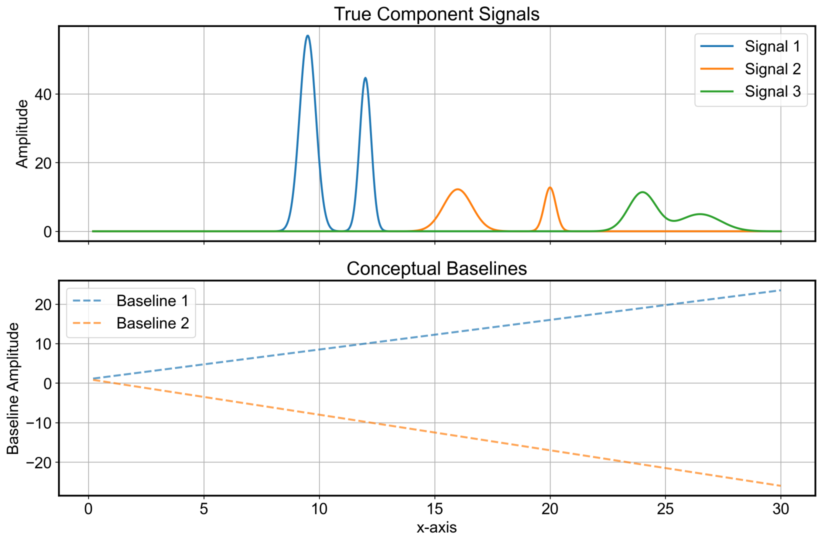

FIG. S3: Description of the artificial data used for testing. True signals, each having two Gaussian peaks, (in top). Linear baseline shown in bottom.

The three signals were mixed with predefined coefficients so as to model spectral mixing as observed in real signals. Linear baseline was added to half of the dataset. The so obtained dataset was 2D, hereby labeled as `true_data` with dimension of 900 (rows) $\times$ 200 (columns) with x-dimension spanning from 0.2 to 30.

As a reference, the first column was fit using least-squares minimization using a convolution of 6 Gaussian functions. The obtained fit parameters were used as reference for a later comparison. Thus, we have following set of parameters for our testing : peak fit parameters and coefficients governing magnitude of Signal 1, Signal 2 and Signal 3 in the one of the vectors of the dataset. These are summarized in Table S4.

Next, noise of varying magnitude was added to the dataset (labeled as `test_data`). This noise was centered at zero and the magnitude of noise is defined using standard deviation (sdev). This was followed by LRA-reconstruction using ranks from 3–10. For each rank, the obtained reconstructed dataset was analyzed as done for the `true_data`, for the parameters listed in Table S4. The used programs are available online.[7] The results are visualized below :

TABLE S4: Analyzed parameters

**Parameter tested**	**Definition**
Peak height	A in $A \cdot \exp\left(-\frac{(x-x_0)^2}{2\sigma^2}\right)$
Peak width	σ in $A \cdot \exp\left(-\frac{(x-x_0)^2}{2\sigma^2}\right)$
Peak area	$A \cdot \sigma \cdot \sqrt{2\pi}$
SNR	Signal / Noise where, Signal = Peak height from baseline ; Noise = 1 σ std devn. from $x = 1.2$ to 7.8
Coefs: c_1, c_2, c_3 representing concentrations	$c_1 \cdot S_1 + c_2 \cdot S_2 + c_3 \cdot S_3$ ($S_1,\ S_2,\ S_3$ are visualized in Fig. S3)

1. Noise magnitude sDev = 15

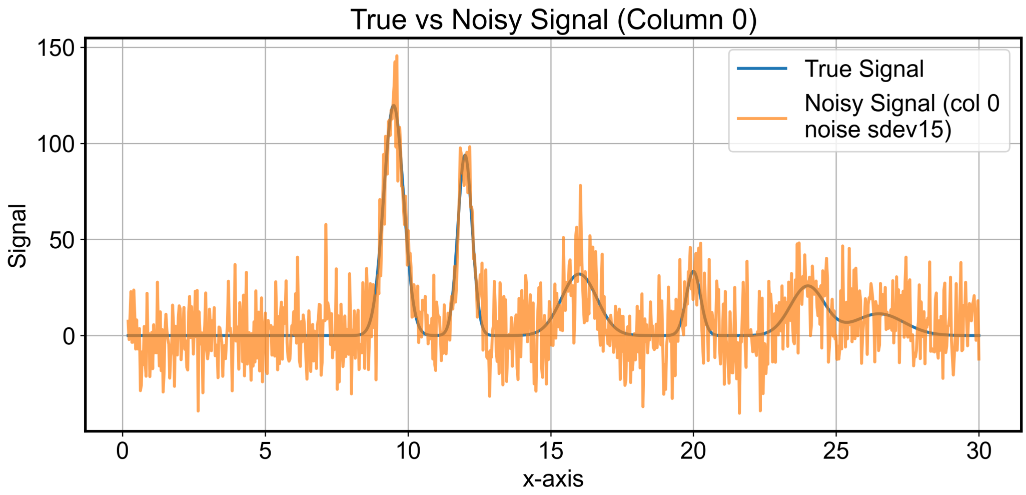

FIG. S5: Column 0 of the true and test dataset. For test dataset, the noise level was 15 (centered at zero).

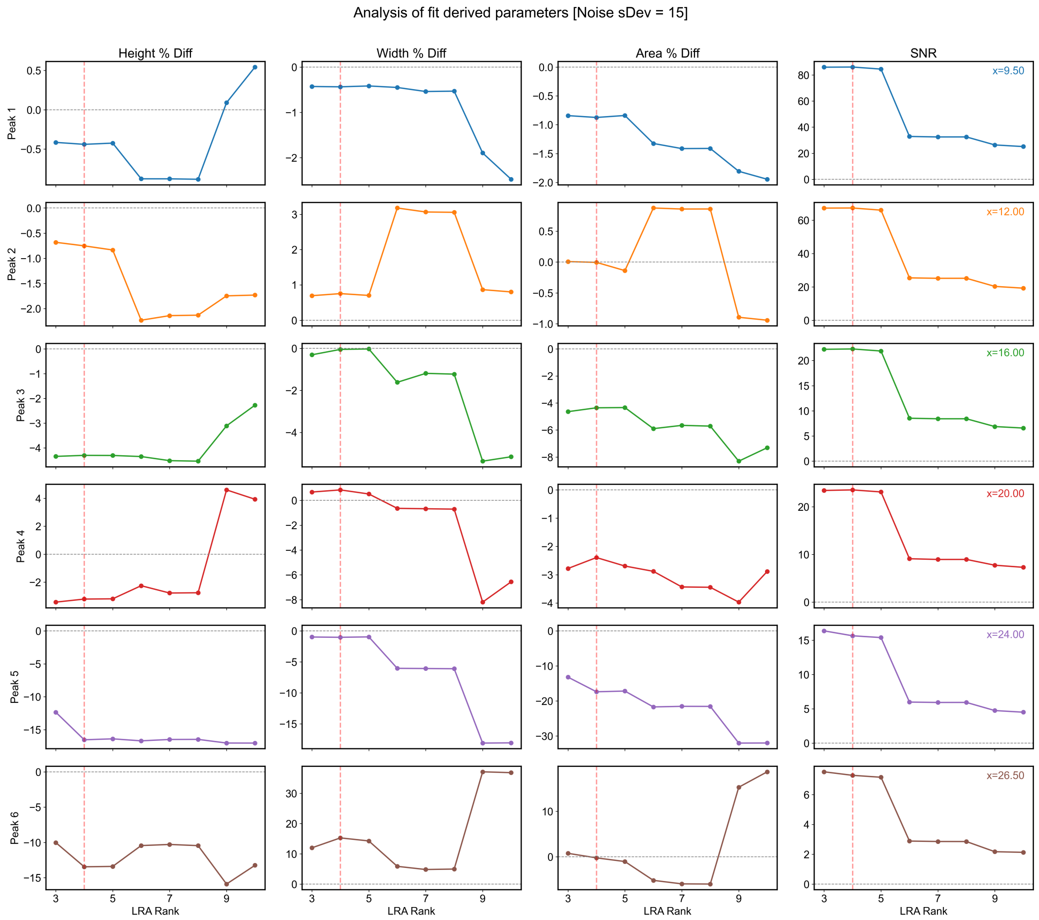

FIG. S6: Plots showing the deviation in the values of peak parameters (arranged horizontally), obtained for the 6 peaks (shown vertically) as a function of the LRA-rank. The standard deviation of the added noise was 15. Cutoff for LRA approximation obtained from SNR > 1 for U_i is shown as dashed red vertical line.

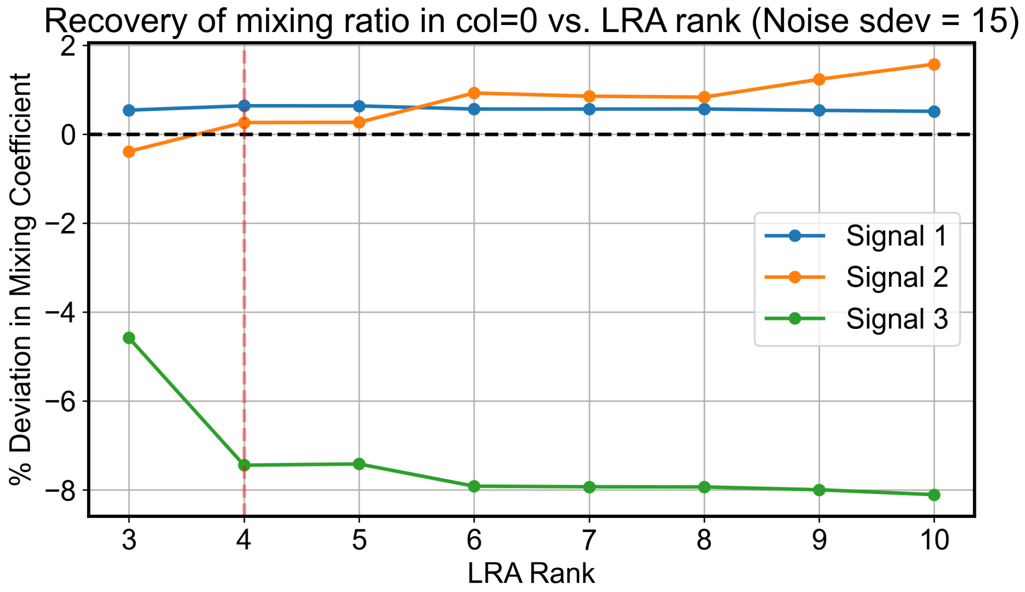

FIG. S7: Concentration of original signals 1–3 (or their mixing ratio) in the noisy dataset (sDev = 15) after LRA approximation. Cutoff for LRA approximation obtained from SNR > 1 for U_i is shown as dashed red vertical line.

2. Noise magnitude sDev = 30

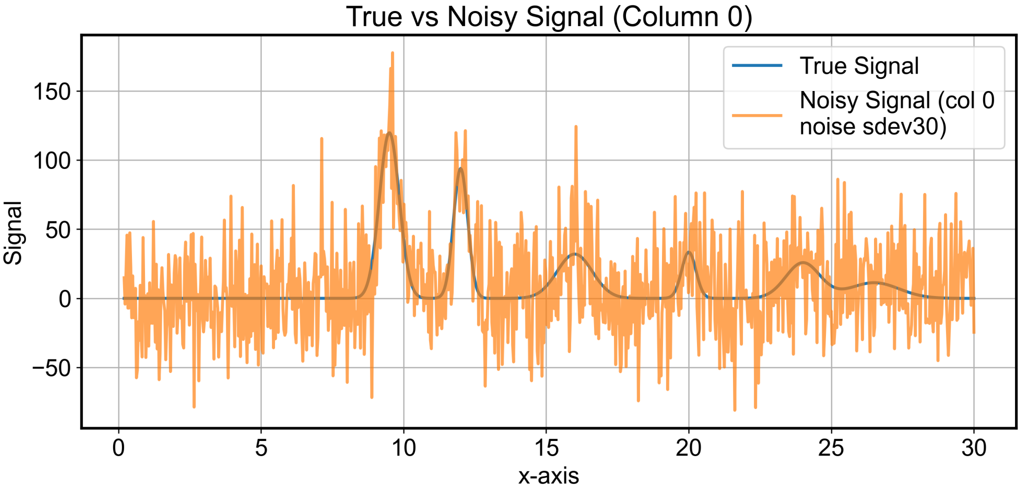

FIG. S8: Column 0 of the true and test dataset. For test dataset, noise level was 15.

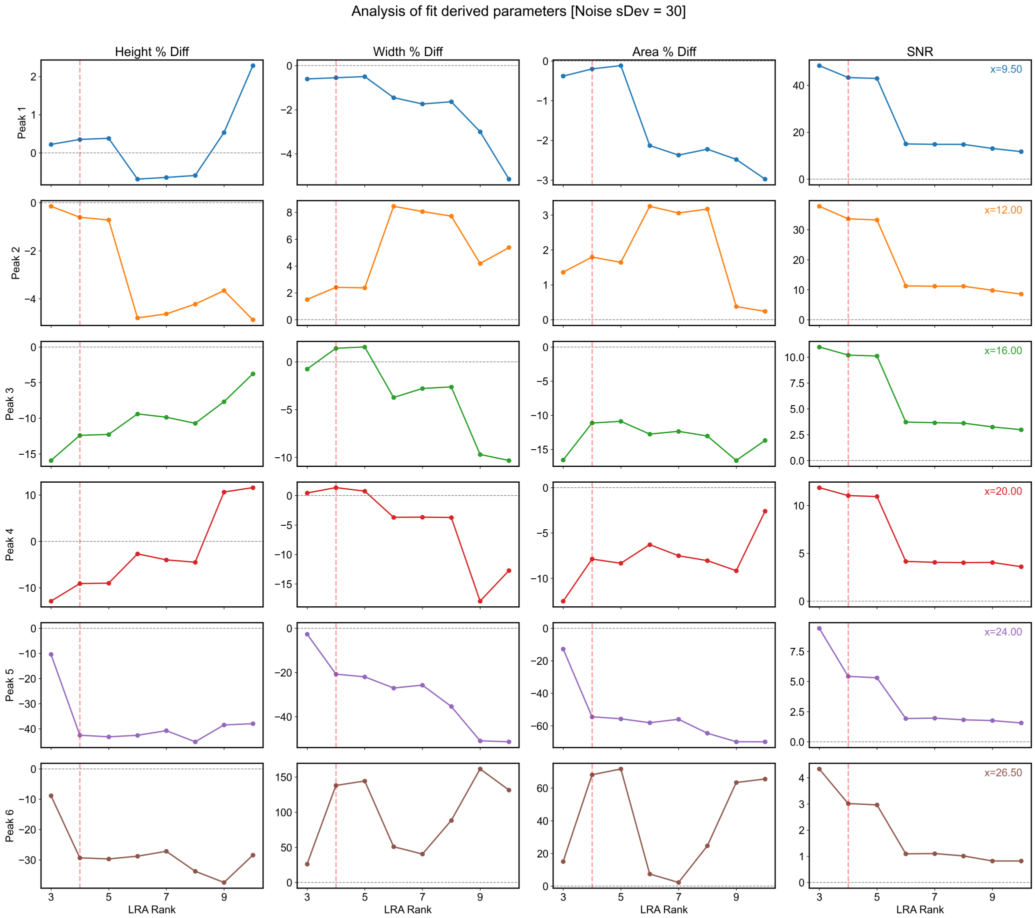

FIG. S9: Plots showing the deviation in the values of peak parameters (arranged horizontally), obtained for the 6 peaks (shown vertically) as a function of the LRA-rank. The standard deviation of the added noise was 30. Cutoff for LRA approximation obtained from SNR > 1 for U_i is shown as dashed red vertical line.

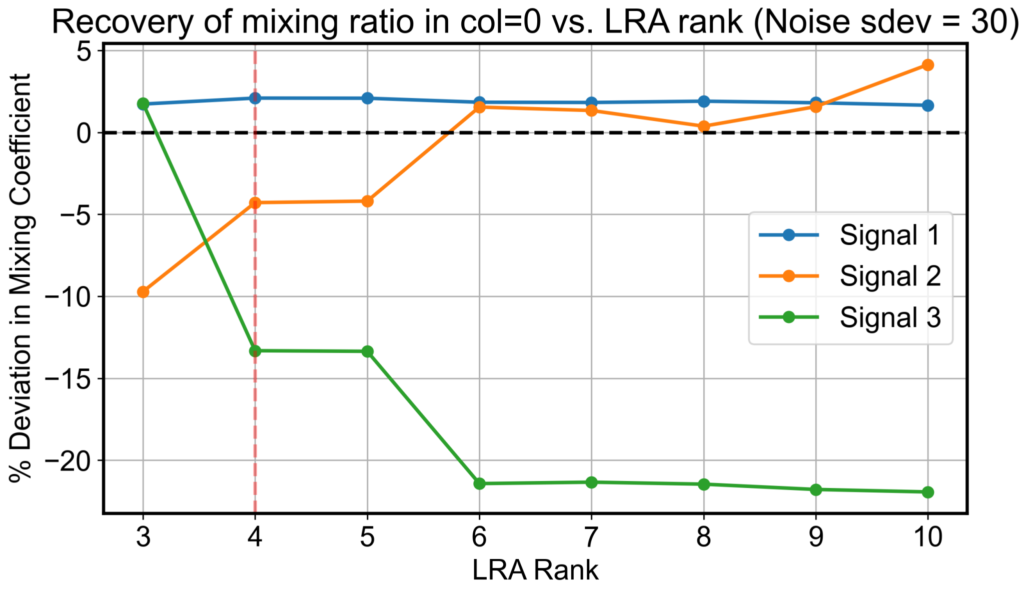

FIG. S10: Concentration of original signals 1–3 (or their mixing ratio) in the noisy dataset (sDev = 15) after LRA approximation. Cutoff for LRA approximation obtained from SNR > 1 for U_i is shown as dashed red vertical line.

3. Summary

1. Peak parameters : In both of the noisy datasets, the obtained fit parameters (height, width and area) showed increasing deviation as the rank increases.
2. Signal-to-noise ratio (SNR) : With larger rank of LRA, the SNR in the obtained dataset worsens.
3. Concentration (mixing ratio) : The obtained concentration profile becomes inaccurate when higher rank for LRA is used.
4. LRA Cutoff, SNR > 1 for U_i : The cutoff for LRA obtained from this testing shows that a good balance between accuracy (in terms of spectral information) and noise-reduction is obtained. This is highlighted by observing the rank $\pm$1 to the one delivered by SNR > 1 for U_i (in this case 4). Cutoff of 3 has better SNR but more erroneous peak parameters (particularly for less strong features), and the vice-versa for rank 5.

From this testing it becomes clear (and what is already known) that retaining more number of spectral components reduces the noise-reduction performance of LRA. Hence, a proper cutoff for retention is required. The present method is based on SNR > 1 for U_i which aims to retain all distinguishable Raman spectral features in the dataset, and prevent information loss.

SM3 Results on the low-rank approximation based denoising of Raman dataset : Cell 1

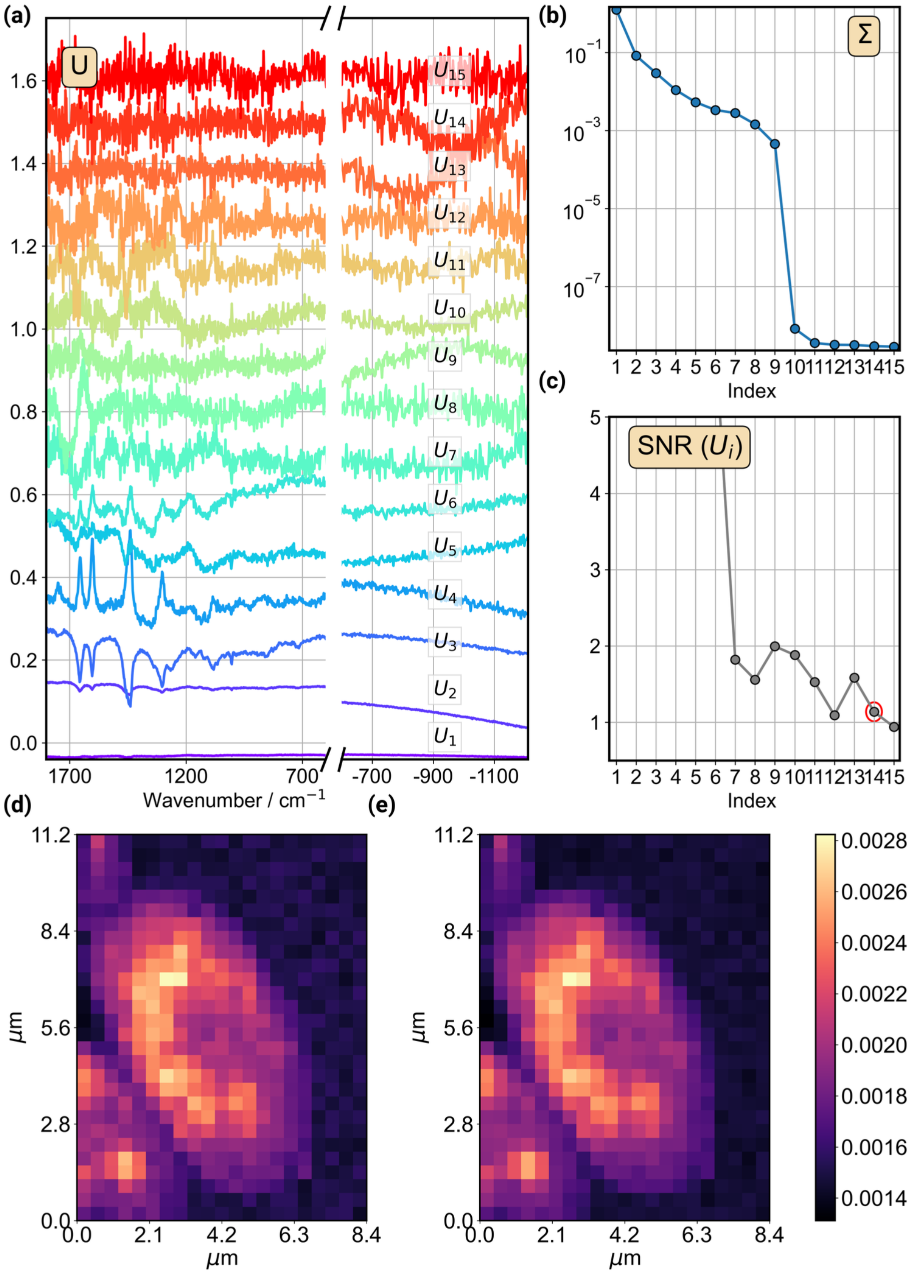

FIG. S11: Results of the SVD denoising process. (a) Top 15 resolved spectral vectors (or basis vectors, (U_i) after SVD analysis of the Raman dataset, (b) Singular values of the corresponding spectral vectors, Σ_i, (c) Signal-to-noise ratio of the spectral vectors computed as per Eqn. (1) in main document, (d) monovariate Raman image constructed using 1443 cm^{-1} band from the raw dataset, and (e) the analogous image constructed from the denoised dataset.

SM4 Automated baseline determination via protein (signal from phenylalanine at 1003 cm^{-1})

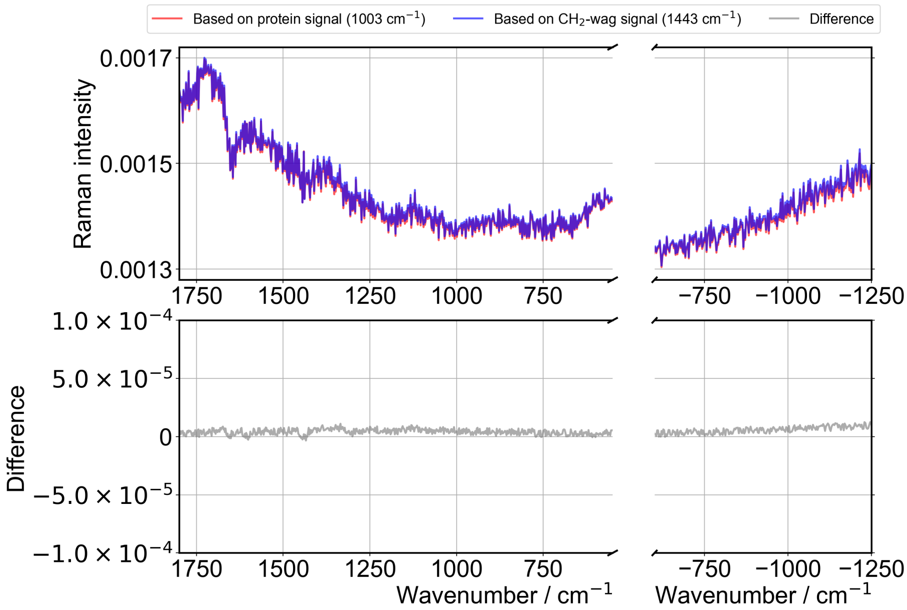

FIG. S12: Baseline determined using the phenylalanine signal at 1003 cm^{-1} (in red) and the one determined with the CH_2 wagging signal at 1443 cm^{-1} (in blue). Their difference, (baseline$_{\text{using 1443}}$ − baseline$_{\text{using 1003}}$) is plotted in grey with expanded y-axis.

SM5 Results on least squares curve-fitting for Raman peak amplitude determination for image construction

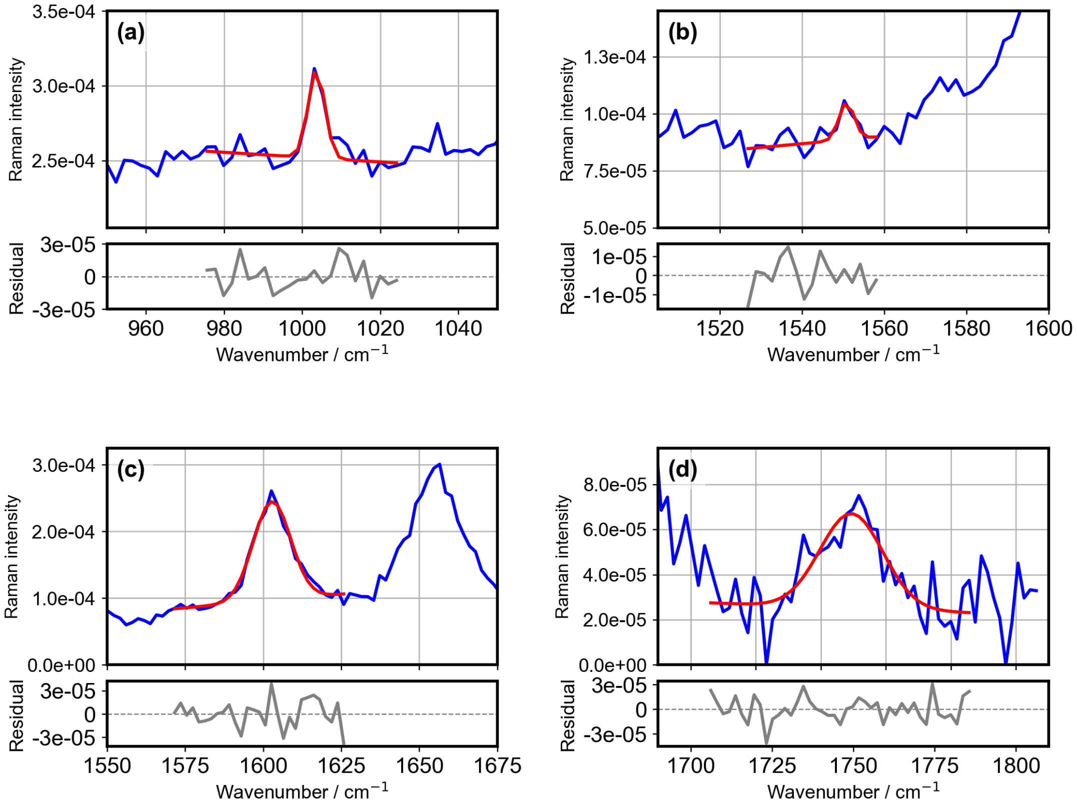

FIG. S13: Peak amplitude determination using curve-fitting for (a) 1003, (b) 1550, (c) 1602 and (d) 1745 cm^{-1} Raman peaks. A linear baseline with a Gaussian function was used together with initial guesses to perform the least-sqaures curve-fit on spectrum of the processed datasets. A representative fit is shown here.

SM6 Comparison of results with other methods of baseline estimation

A Details of baseline estimation methods discussed in main document (in Fig. 9)

Target Raman spectra were truncated to analyze spectral region from 575 cm^{-1} to 1806 cm^{-1}. ASLS[8], ASPLS[9] and MPLS[10] techniques were used as implemented in the `pybaseline` package[11], and used as described below :

```

from pybaselines import Baseline, utils

# truncation
raw_subset = raw[:602]  # numpy 1D array
xaxis_subset = xaxis[:602] # numpy 1D array

print(xaxis_subset[0], xaxis_subset[-1])

baseline_fitter = Baseline(x_data=xaxis_subset )

method = [  'ASLS', 'ASPLS', 'MPLS']

# -----------------
# PARAMETERS
lam = 4e3
tol = 1e-6
max_iter = 500
# -----------------
bkg_asls, params_1 = baseline_fitter.asls(raw_subset, lam=lam,
tol=tol,
max_iter=max_iter)

bkg_aspls, params_2 = baseline_fitter.aspls(raw_subset, lam=lam,
tol=tol,
max_iter=max_iter)

bkg_mpls, params_3 = baseline_fitter.mpls(raw_subset, lam=1e4)
#-----------------------------------------------------------------
```

Code block 2: Usage details of the baseline estimation methods with the parameters

Several values of the parameter `lam` were tested, all of which distorted the resulting subtracted spectra as shown in Fig. 9 of the main document. For additional comparisons see Sec. SM6 B.

For polynomial based baseline modeling, masking was used to limit the fit to spectral region where no distinct Raman features are present. This process is subjective and the result depends on : fit region analysed and the order of polynomial function. Spectral region used for fit (Poly-4 and Poly-5) is illustrated in Fig. S14.

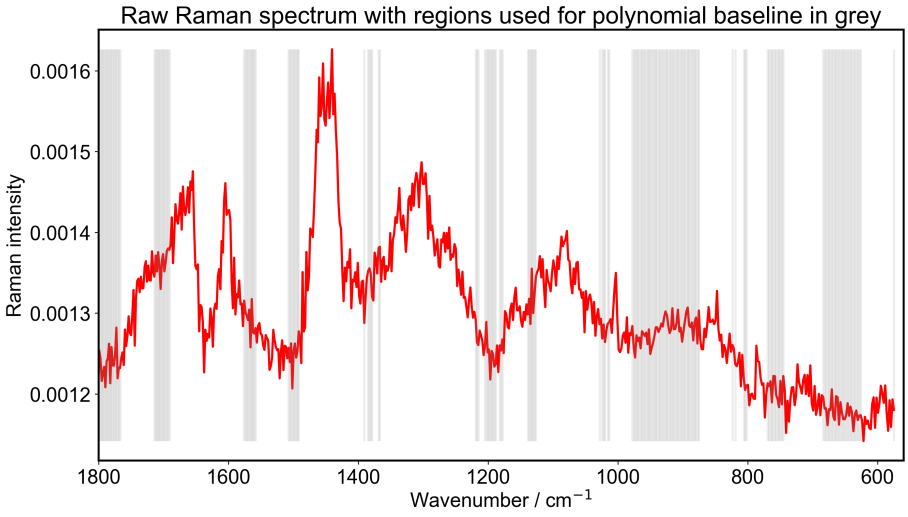

FIG. S14: Raw Raman spectrum together with the masked region used for fitting in grey color.

B Additional comparison

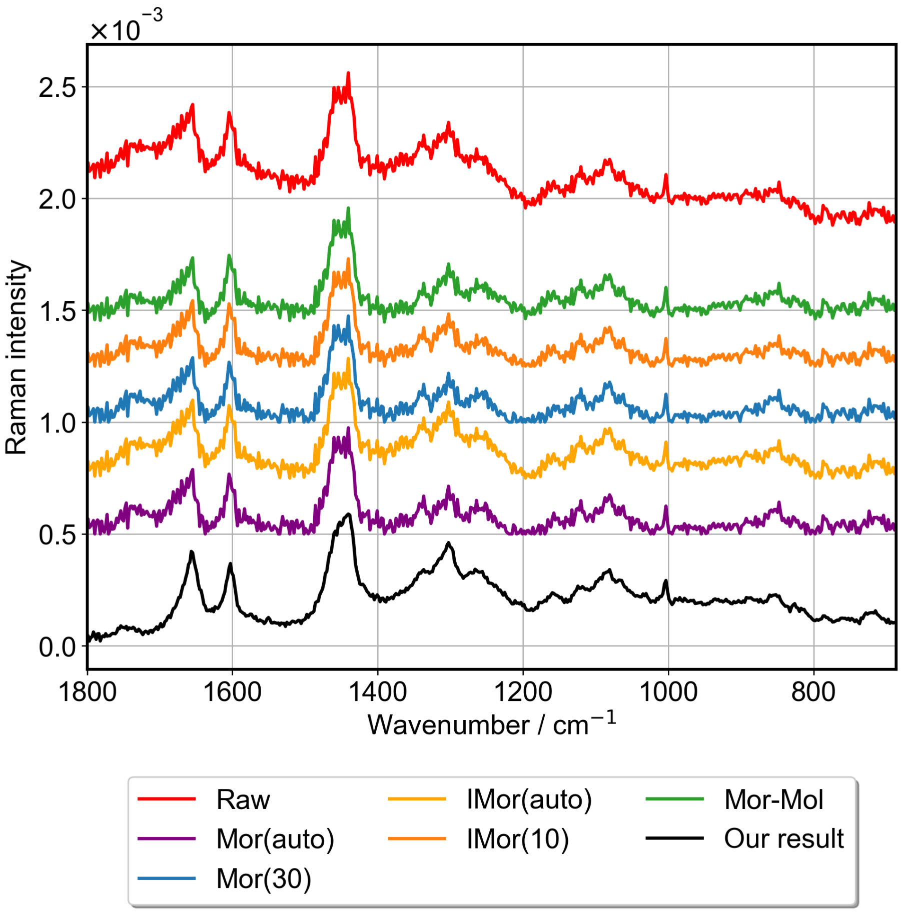

FIG. S15: Comparative evaluation of the Raman background removal via HAMAND in this work, with baseline-estimation from 5 methods (with subtraction using a factor of one). Input spectrum (labeled as ‘Raw‘) is in red. Spectra are shown with an offset to aid in visualization. See details below for the parameters used in this evaluation.

```
from pybaselines import Baseline, utils


raw_subset = raw[:602]
xaxis_subset = xaxis[:602]

print(xaxis_subset[0], xaxis_subset[-1])

baseline_fitter = Baseline(x_data=xaxis_subset )
# -------------------------------------------------------
method_set2 = [ 'Mor(30)', 'Mor(Auto)', 'IMor(30)', 'IMor(Auto)', 'Mor-Mol' ]
# -------------------------------------------------------
# PARAMETER
half_window = 30
# ----------------------




bkg_mor30, params_1 = baseline_fitter.mor(raw_subset, half_window)

# autmatic half window determined internally
bkg_mor_auto, params_1 = baseline_fitter.mor(raw_subset )

bkg_imor, params_1 =  baseline_fitter.imor(raw_subset, half_window)

# autmatic half windoe determined internally
bkg_imor_auto, params_1 =  baseline_fitter.imor(raw_subset)

```

```
bkg_mormol, params_1 = baseline_fitter.mormol(
raw_subset,
half_window,
smooth_half_window=10,
pad_kwargs={'extrapolate_window': 20}      )
# ------------------------------------------------------
```

Code block 3: Usage details of the other baseline estimation methods with the used parameters

SM7 Result of MCR-ALS decomposition

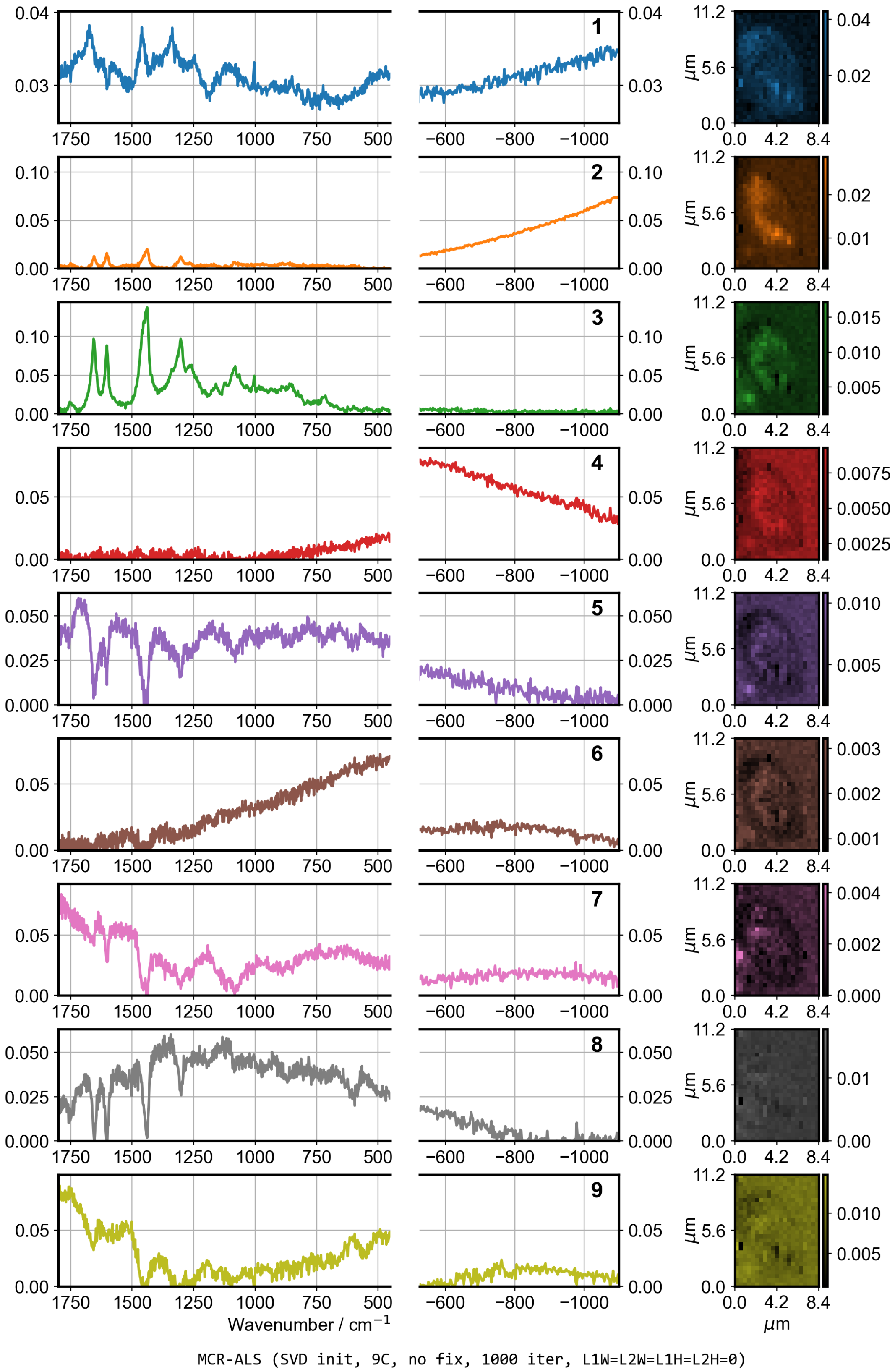

FIG. S16: Results from MCR-ALS analysis assuming 9 components for the Raman dataset from Cell 1.

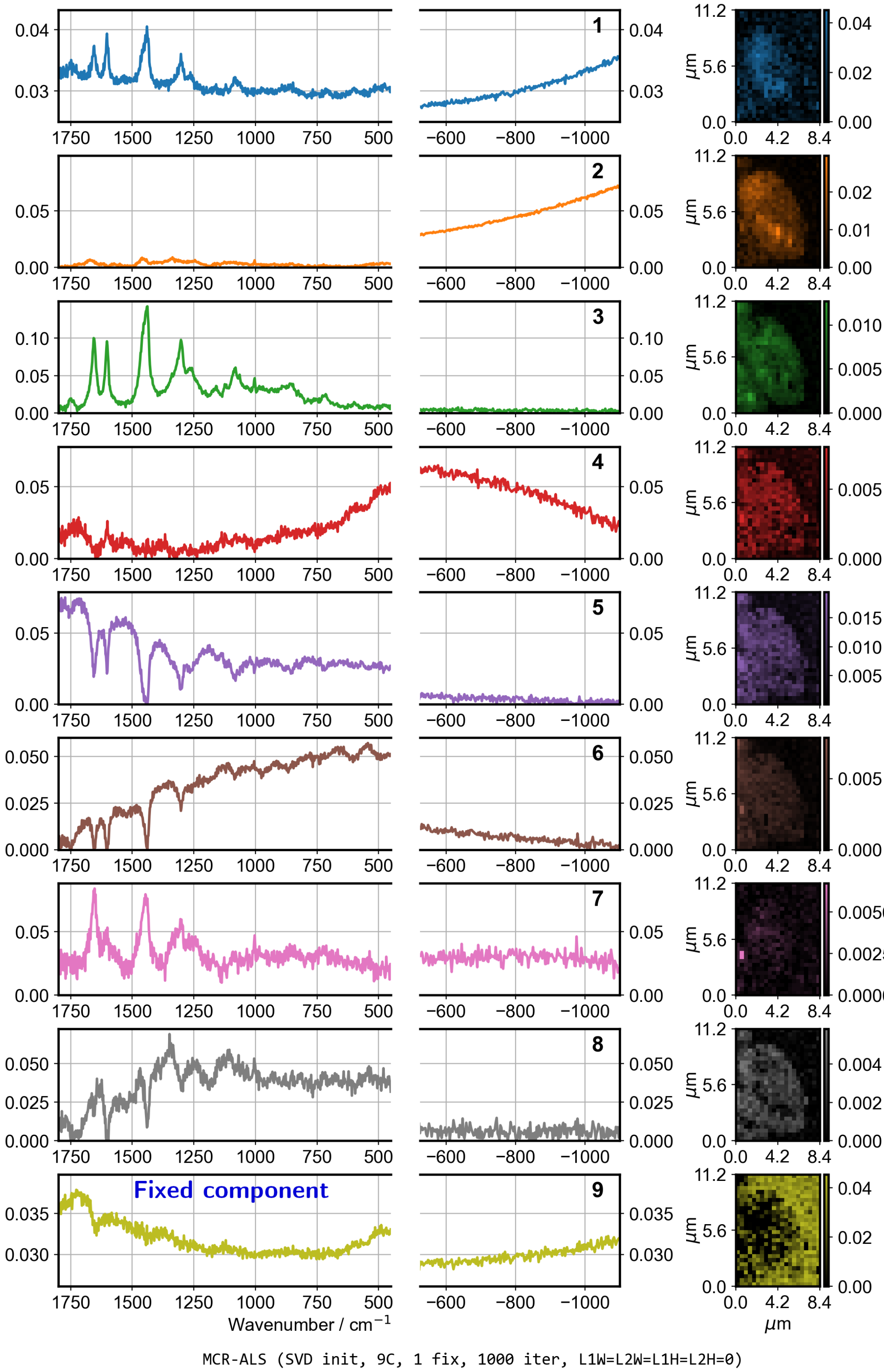

FIG. S17: Results from MCR-ALS analysis assuming 9 components for the Raman dataset from Cell 1. One of the spectral component, the automatically determined background labelled as 9, was fixed in this analysis.

MCR-ALS decompositions[12] using 9 spectral components were tested since SVD analysis showed 9 major components (see Fig. S11 for the SVD result). Initialization vectors were obtained from SVD performed internally. The obtained results show complex mixing of spectral components, indicating an incorrect solution.

SM8 Results from additional *S. pombe* cells

A Cell 2

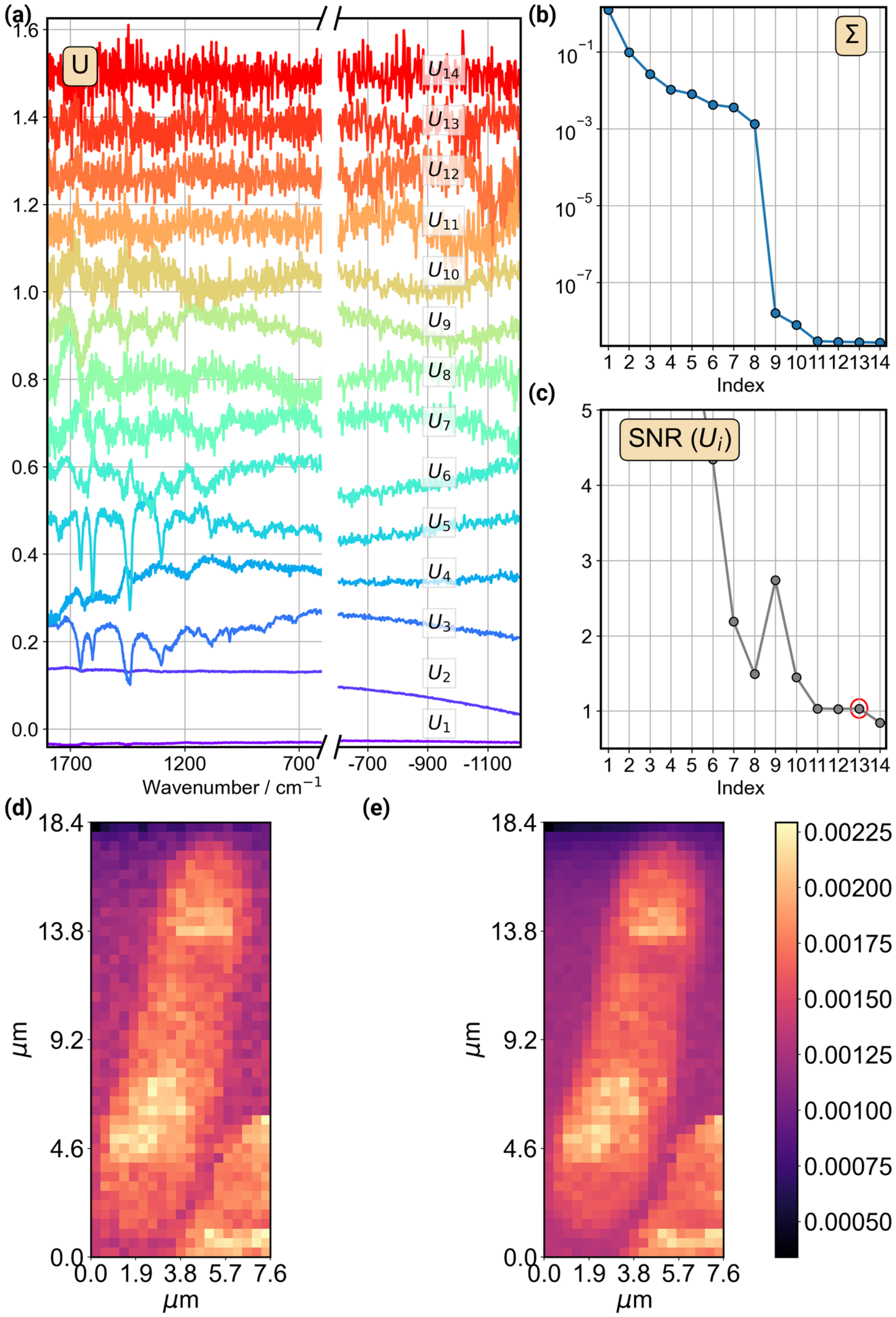

FIG. S18: Result from SVD analysis (and denoising) of the Raman dataset for cell 2. (a) Top 14 resolved spectral vectors (or basis vectors, U_i) after SVD analysis of the Raman dataset, (b) Singular values of the corresponding spectral vectors, Σ_i, (c) Signal-to-noise ratio of the spectral vectors, see Sec. SM2 for details, (d) mono-variate image constructed using 1443 cm^{-1} band from the raw dataset, and (e) the analogous image from the denoised dataset.

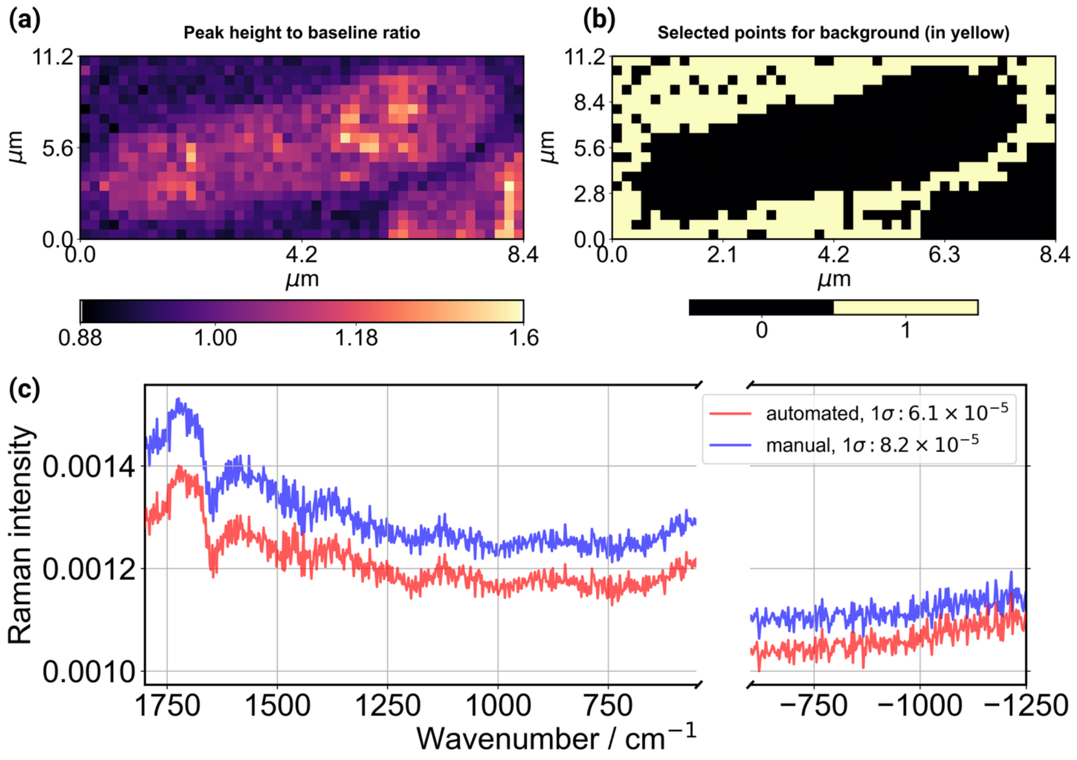

FIG. S19: Peak-height-to-baseline filter for automated determination of the background spectra for cell 2. Sub-figure (a) shows the peak height-to-baseline intensity ratio the specific spectra at each pixel. For points outside the cell, this ratio is very close to unity. Sub-fig (b) shows the spatial location of the selected spectra from outside the cell in yellow, and the points from inside the cell in black color. Sub-fig (c) shows the averaged background spectrum from the automated analysis in red color, along with averaged background spectrum from manual selection of 10 points from outside the cell (in blue color) and the 1σ standard deviation is indicated.

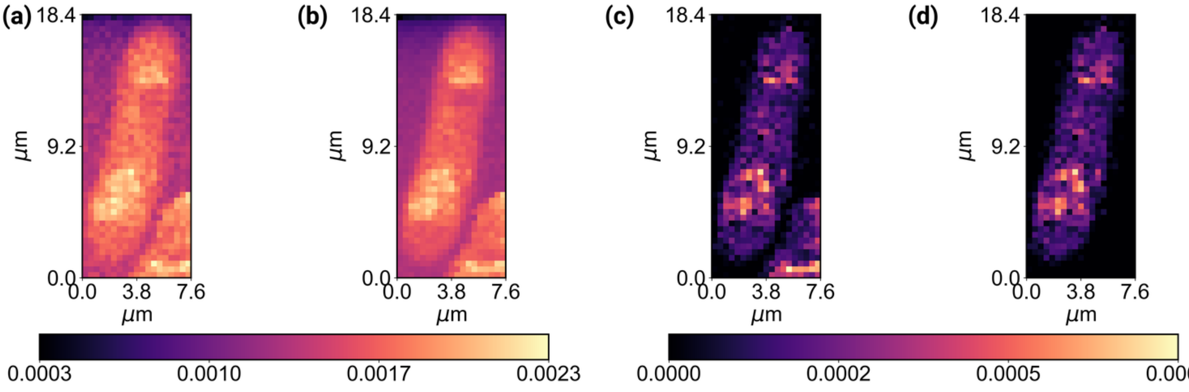

FIG. S20: Summary of the analysis for cell 2. (a) Mono-variate Raman image constructed using the Raman peak at 1443 cm^{-1} from raw Raman dataset and analogous image constructed using the denoised dataset in (b), image constructed after subtraction of the automatically determined background in (c), and (d) the analogous image after spectral cleanup to remove portion of cells.

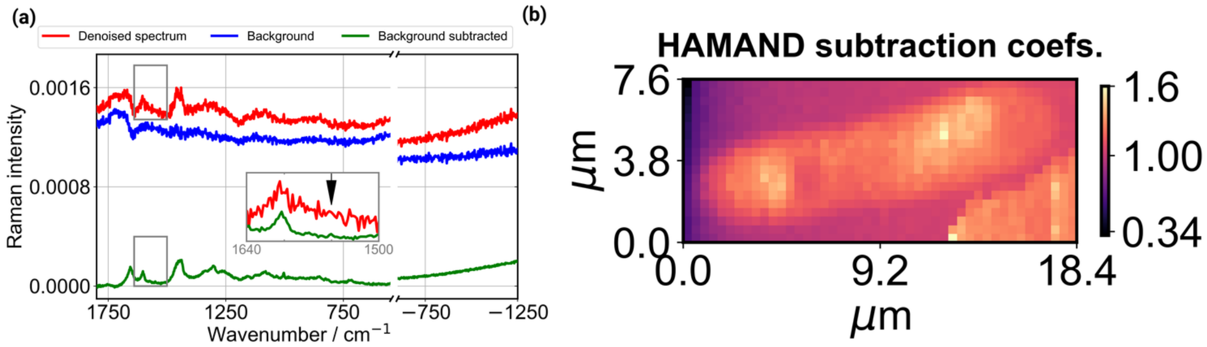

FIG. S21: (a) Raman spectra from a point inside the *S. pombe* cell-2 illustrating the effect of data analysis process. The spectrum after LRA based denoising is shown in red. The background spectrum, determined automatically, is in blue, and lastly, the final spectra after subtracting the background using HAMAND is in green. The 1550 cm^{-1} peak is discernible from noise only after subtraction of the background. Subtraction coefficient determined by HAMAND is visualized in subplot (b)

B Cell 3

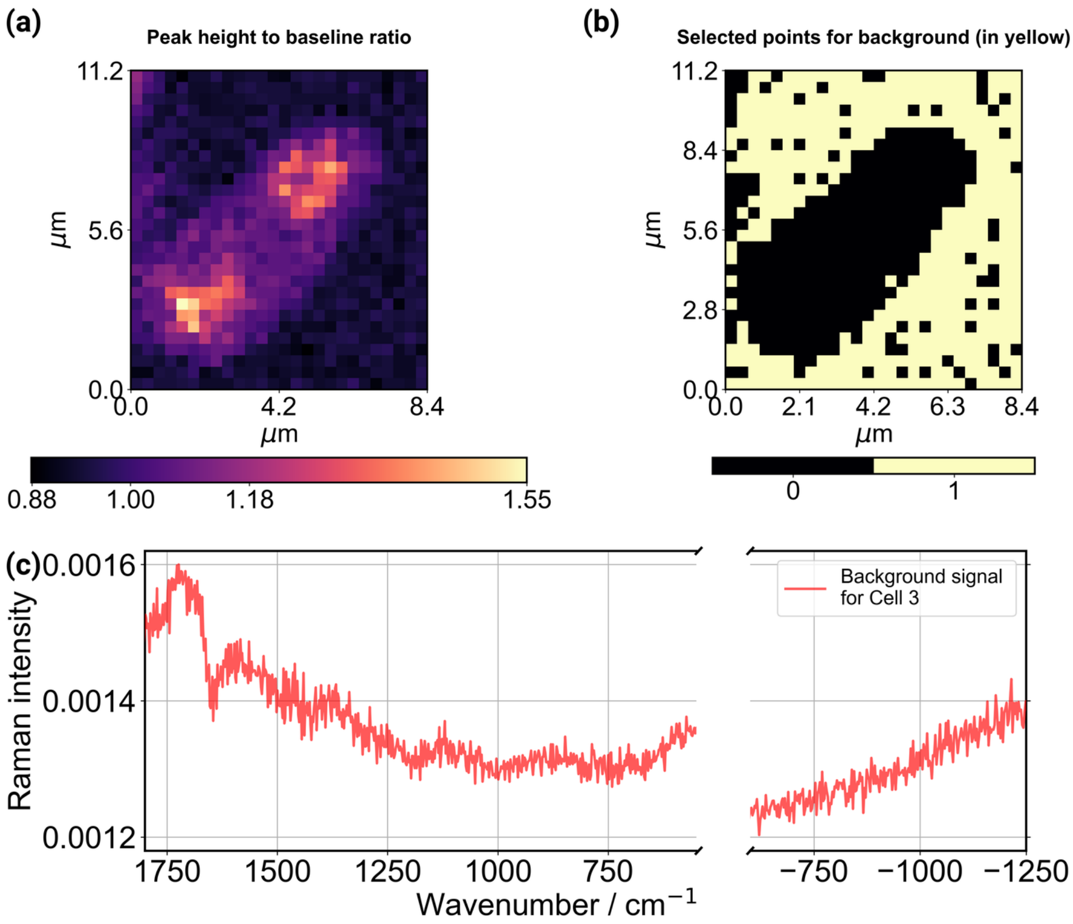

FIG. S22: Peak-height-to-baseline filter for automated determination of the background spectra for cell 3. Sub-figure (a) shows the peak height-to-baseline intensity ratio the specific spectra at each pixel. For points outside the cell, this ratio is very close to unity. Sub-fig (b) shows the spatial location of the selected spectra from outside the cell in yellow, and the points from inside the cell in black. Sub-fig (c) shows the obtained averaged background spectrum.

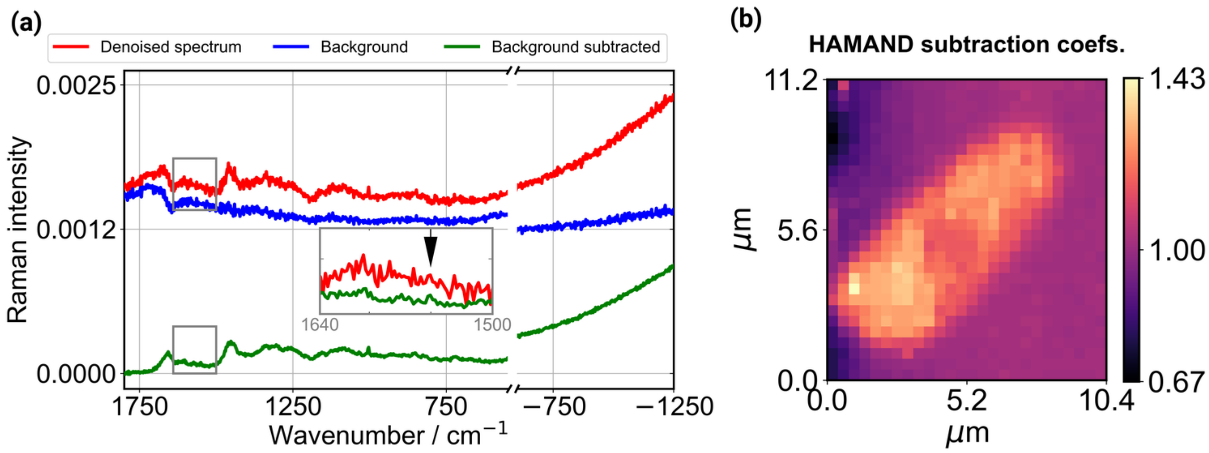

FIG. S23: (a) Raman spectra from a point inside cell-3 illustrating the effect of the data analysis process. The spectrum after LRA based denoising is shown in red. The background spectrum, determined automatically, is in blue, and lastly, the final spectra after subtracting the background using HAMAND is in green. The 1550 cm^{-1} peak is discernible from noise only after subtraction of the background. Subtraction coefficient determined by HAMAND is visualized in subplot (b)

C Cell 4

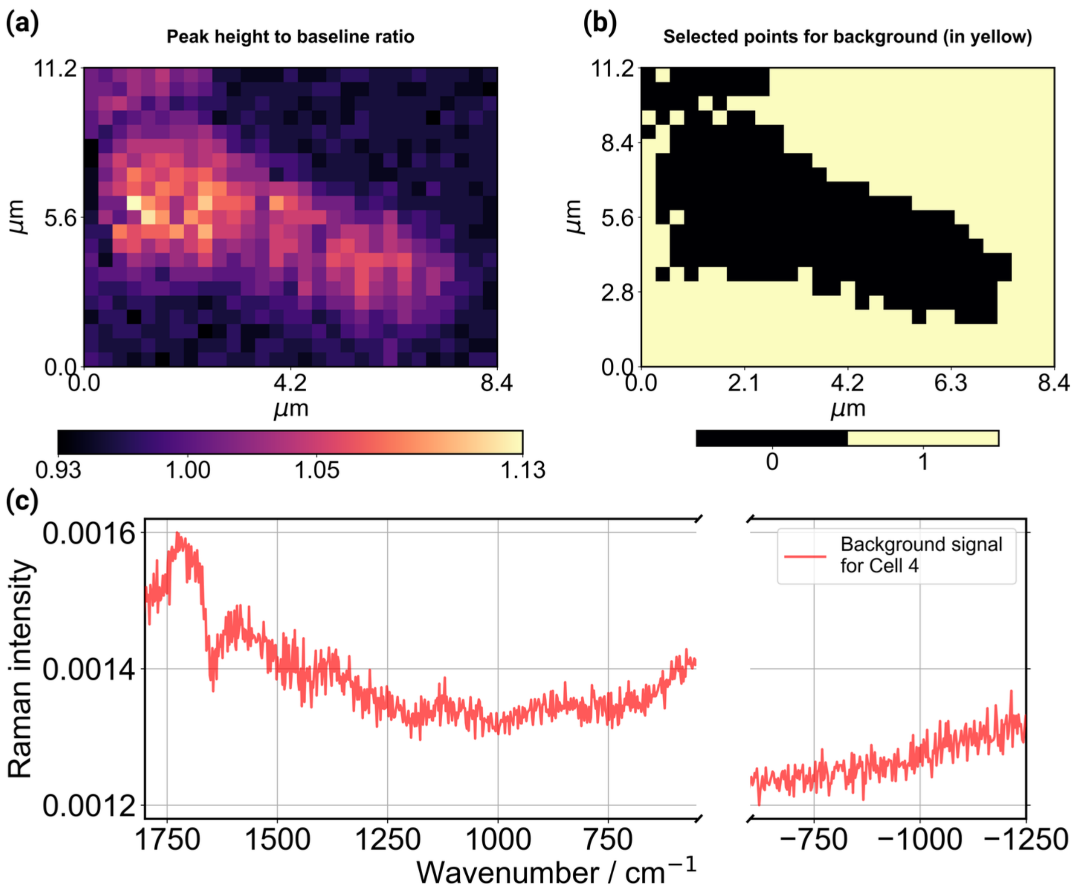

FIG. S24: Peak-height-to-baseline filter for automated determination of the background spectra for cell 4. Sub-figure (a) shows the peak height-to-baseline intensity ratio the specific spectra at each pixel. For points outside the cell, this ratio is very close to unity. Sub-fig (b) shows the spatial location of the selected spectra from outside the cell in yellow, and the points from inside the cell in black. Sub-fig (c) shows the obtained averaged background spectrum.

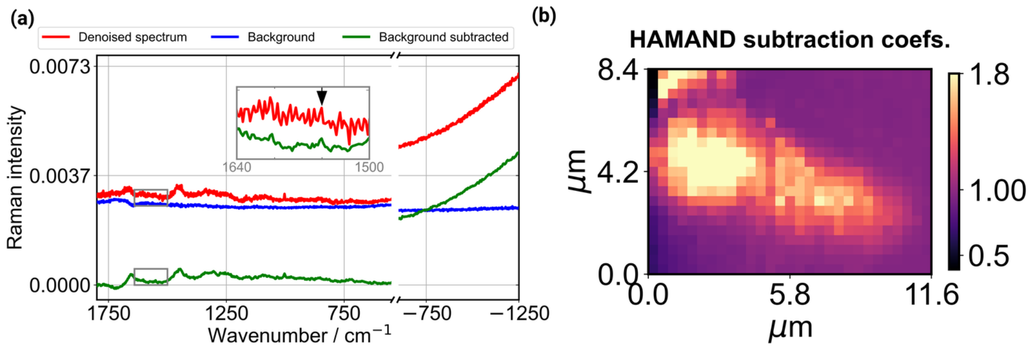

FIG. S25: (a) Raman spectra from a point inside cell-4 illustrating the effect of the data analysis process. The spectrum after LRA based denoising is shown in red. The background spectrum, determined automatically, is in blue, and lastly, the final spectra after subtracting the background using HAMAND is in green. The 1550 cm^{-1} peak is discernible from noise only after subtraction of the background. Subtraction coefficients determined by HAMAND are visualized in subplot (b).

SM9 Focal point change with refractive index of the medium

Fig. S26 shows the path of a focused excitation beam (in red) inside a uniform medium, in the present employed back-scattering geometry.

The refractive index of immersion oil, cover-slip and aqueous medium are marked as n_{Oil}, n_Q and n_s, respectively. The angle subtended by the converging beam at the quartz surface is given by θ_1, which is determined using the numerical aperture and refractive index of the immersion oil (see details later).

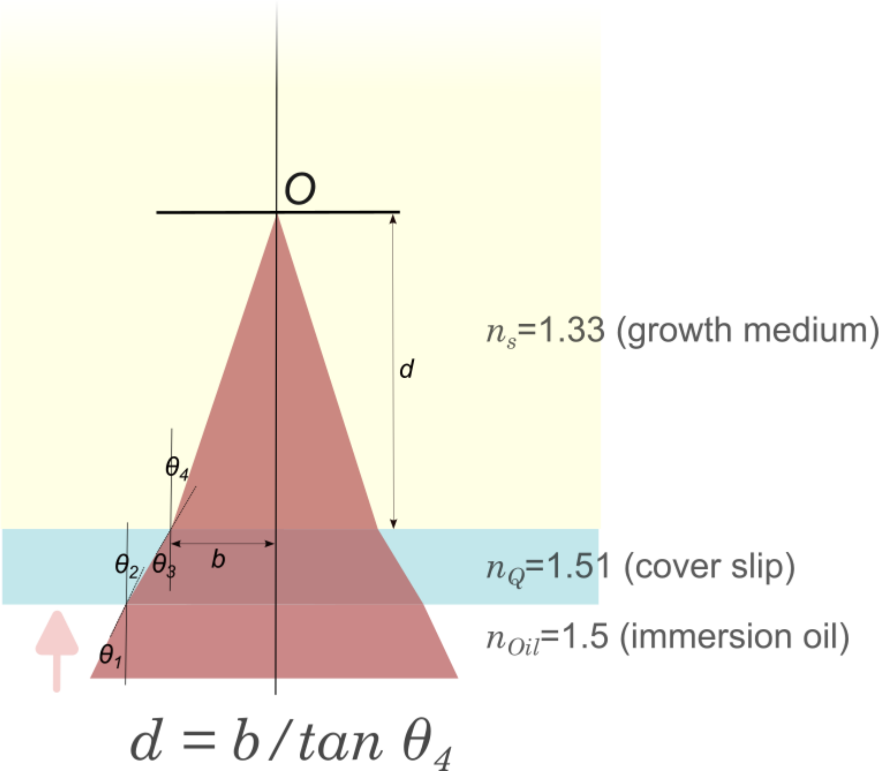

FIG. S26: Back-scattering geometry is used in the present work, where an objective lens is used for excitation and collection. Illustration shows the movement of converging excitation beam inside the sample while undergoing refraction at the interfaces. Propagation of light at the immersion oil-coverslip interface, and the coverslip-liquid (sample) interface is illustrated.

Angle subtended by the incident beam while propagating inside the sample is determined using Snell's law, as a function of the refractive indices of the medium,

$$\theta_4 = \sin^{-1}\left(\frac{n_Q}{n_s} \cdot \sin\theta_2\right), \tag{S27}$$

where θ_2 is given by

$$\theta_2 = \sin^{-1}\left(\frac{n_{Oil}}{n_Q} \cdot \sin\theta_1\right), \tag{S28}$$

where θ_1 is determined by the known NA and refractive index of the medium (immersion oil) as $\theta_1 = sin^{-1}(NA/n_{Oil})$. The angle, $\theta_1 =$ was computed to 1.102 rad (59°) for the presently used 100$\times$ oil immersion lens. Lastly, magnitude of θ_4 changes with refractive index of the the sample, n_s, (shown as yellow region in Fig. S26).

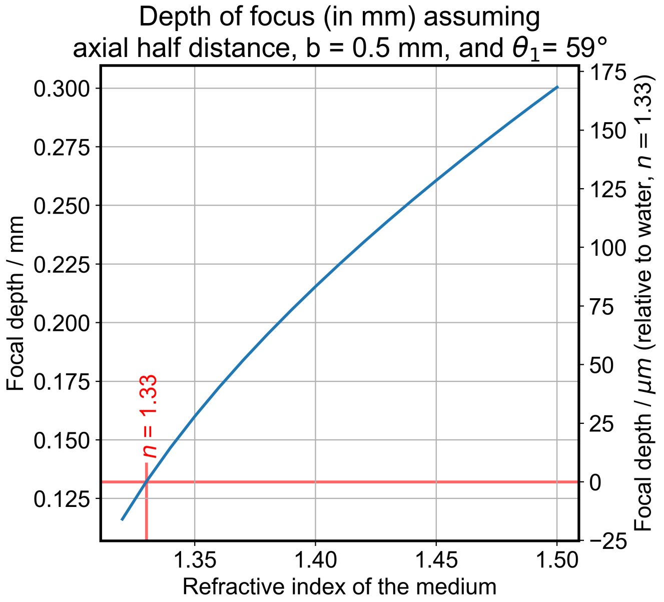

FIG. S29: Change in the focal depth modelled using geometrical optics assuming point focus.

With θ_4 known using Eqn. S27 and axial length, b, representing radius of the circular beam on the quartz-sample interface, the depth inside the sample is computed as $d = b/\tan\theta_4$. This treatment assumes that b is large compared to the focused-beam waist at the focal spot. Calculated focal depth with different refractive indices from 1.32 to 1.51 is shown in Fig. S29 illustrating the variation of depth of focal spot.

Bibliography

[1] K. Ekwall and G. Thon, Setting up Schizosaccharomyces pombe crosses/matings, Cold Spring Harbor Protocols **2017**, pdb.prot091694 (2017).

[2] C. Eckart and G. Young, The approximation of one matrix by another of lower rank, Psychometrika **1**, 211–218 (1936).

[3] G. H. Golub and C. F. Van Loan, *Matrix Computations*, 3rd ed. (The Johns Hopkins University Press, 1996).

[4] A. Savitzky and M. J. E. Golay, Smoothing and differentiation of data by simplified least squares procedures, Anal. Chem. **36**, 1627 (1964).

[5] P. Virtanen, R. Gommers, T. E. Oliphant, M. Haberland, T. Reddy, D. Cournapeau, E. Burovski, P. Peterson, W. Weckesser, J. Bright, S. J. van der Walt, M. Brett, J. Wilson, K. J. Millman, N. Mayorov, A. R. J. Nelson, E. Jones, R. Kern, E. Larson, C. J. Carey, İ. Polat, Y. Feng, E. W. Moore, J. VanderPlas, D. Laxalde, J. Perktold, R. Cimrman, I. Henriksen, E. A. Quintero, C. R. Harris, A. M. Archibald, A. H. Ribeiro, F. Pedregosa, P. van Mulbregt, A. Vijaykumar, A. P. Bardelli, A. Rothberg, A. Hilboll, A. Kloeckner, A. Scopatz, A. Lee, A. Rokem, C. N. Woods, C. Fulton, C. Masson, C. Häggström, C. Fitzgerald, D. A. Nicholson, D. R. Hagen, D. V. Pasechnik, E. Olivetti, E. Martin, E. Wieser, F. Silva, F. Lenders, F. Wilhelm, G. Young, G. A. Price, G.-L. Ingold, G. E. Allen, G. R. Lee, H. Audren, I. Probst, J. P. Dietrich, J. Silterra, J. T. Webber, J. Slavič, J. Nothman, J. Buchner, J. Kulick, J. L. Schönberger, J. V. de Miranda Cardoso, J. Reimer, J. Harrington, J. L. C. Rodríguez, J. Nunez-Iglesias, J. Kuczynski, K. Tritz, M. Thoma, M. Newville, M. Kümmerer, M. Bolingbroke, M. Tartre, M. Pak, N. J. Smith, N. Nowaczyk, N. Shebanov, O. Pavlyk, P. A. Brodtkorb, P. Lee, R. T. McGibbon, R. Feldbauer, S. Lewis, S. Tygier, S. Sievert, S. Vigna, S. Peterson, S. More, T. Pudlik, T. Oshima, T. J. Pingel, T. P. Robitaille, T. Spura, T. R. Jones, T. Cera, T. Leslie, T. Zito, T. Krauss, U. Upadhyay, Y. O. Halchenko, and Y. V. Baeza, SciPy 1.0: fundamental algorithms for scientific computing in python, Nature Methods **17**, 261 (2020).

[6] Igor Pro, WaveMetrics, Lake Oswego, OR, USA, A scientific data analysis software with numerical computing environment and a programming language.

[7] A. Raj, LRA-analysis: Testing lra denoising for spectroscopic dataset with regard to peak parameters.

[8] P. H. C. Eilers, A perfect smoother, Analytical Chemistry **75**, 3631 (2003).

[9] S.-J. Baek, A. Park, Y.-J. Ahn, and J. Choo, Baseline correction using asymmetrically reweighted penalized least squares smoothing, Analyst **140**, 250 (2015).

[10] Z. Li, D.-J. Zhan, J.-J. Wang, J. Huang, Q.-S. Xu, Z.-M. Zhang, Y.-B. Zheng, Y.-Z. Liang, and H. Wang, Morphological weighted penalized least squares for background correction, Analyst **138**, 4483 (2013).
[11] D. Erb, pybaselines: A Python library of algorithms for the baseline correction of experimental data.
[12] S. Rutan, A. de Juan, and R. Tauler, 2.15 - Introduction to Multivariate Curve Resolution, in *Comprehensive Chemometrics*, edited by S. D. Brown, R. Tauler, and B. Walczak (Elsevier, Oxford, 2009) pp. 249–259.
